# AtCSLD3 and GhCSLD3 mediate root growth and cell elongation downstream of the ethylene response pathway in Arabidopsis

**DOI:** 10.1093/jxb/erx470

**Published:** 2017-12-14

**Authors:** Huizhen Hu, Ran Zhang, Shuchao Dong, Ying Li, Chunfen Fan, Yanting Wang, Tao Xia, Peng Chen, Lingqiang Wang, Shengqiu Feng, Staffan Persson, Liangcai Peng

**Affiliations:** 1 Biomass and Bioenergy Research Centre, Huazhong Agricultural University, China; 2 National Key Laboratory of Crop Genetic Improvement, Huazhong Agricultural University, China; 3 College of Plant Science and Technology, Huazhong Agricultural University, China; 4 College of Life Science and Technology, Huazhong Agricultural University, China; 5 School of Biosciences, University of Melbourne, Australia

**Keywords:** *AtCSLD3*, *GhCSLD3*, cellulose synthesis, cell elongation, ethylene response, phosphate starvation, root growth

## Abstract

*CSLD3*, a gene of the cellulose synthase-like D family, affects root hair elongation, but its interactions with ethylene signaling and phosphate-starvation are poorly understood. Here, we aim to understand the role of CSLD3 in the context of the ethylene signaling and phosphate starvation pathways in Arabidopsis plant growth. Therefore, we performed a comparative analysis of the *csld3-1* mutant, *CSLD3*-overexpressing lines, and ethylene-response mutants, such as the constitutive ethylene-response mutant *i-ctr1*. We found that *CSLD3* overexpression enhanced root and hypocotyl growth by increasing cell elongation, and that the root growth was highly sensitive to ethylene treatment (1 µM ACC), in particular under phosphate starvation. However, the CSLD3-mediated hypocotyl elongation occurred independently of the ethylene signaling pathway. Notably, the typical induction of root hair and root elongation by ethylene and phosphate-starvation was completely abolished in the *csld3-1* mutant. Furthermore, *i-ctr1 csld3-1* double-mutants were hairless like the *csld3-1* parent, confirming that CSLD3 acts downstream of the ethylene signaling pathway during root growth. Moreover, the CSLD3 levels positively correlated with cellulose levels, indicating a role of CSLD3 in cellulose synthesis, which may explain the observed growth effects. Our results establish how CSLD3 works in the context of the ethylene signaling and phosphate-starvation pathways during root hair growth, cell elongation, and cell wall biosynthesis.

## Teaser

CSLD3 overexpression enhances root and hypocotyl growth by increasing cell elongation; CSLD3-mediated hypocotyl elongation is independent of ethylene signaling.

## Introduction

Root hairs are filamentous cells that grow in a defined direction by depositing flexible cell wall material at one site of the cell and exploiting turgor pressure as the driving force for elongation; a process also referred to as tip growth. The root hairs develop and emerge from root epidermal cells, termed trichoblasts (Dolan and Davies, 2004). In Arabidopsis, a root hair develops in a stepwise manner divided into three phases: cell specification, initiation, and elongation (tip growth) (Cho and Cosgrove, 2002). Root hairs are thus important for studies of cell differentiation, development, and elongation (Kacprzyk *et al.*, 2014; Li *et al.*, 2014).

Root hair development is influenced by a plethora of internal and external factors (Grierson and Schiefelbein, 2002; Wada *et al.*, 2015). In particular, soil nutrients [including phosphorus (P), nitrogen (N), iron (Fe), potassium (K), manganese (Mn), and magnesium (Mg)] impact on root hair growth (Müller and Schmidt, 2004; Shin *et al.*, 2005; Yang *et al.*, 2008; Jung *et al.*, 2009; Bloch *et al.*, 2011). For example, plants assimilate P in the form of phosphate (Pi) that typically promotes primary root growth in plants, including Arabidopsis, maize, rice, and tomato. In contrast, Pi starvation increases the production of root hairs and lateral root growth (López-Arredondo *et al.*, 2014). This response increases the ability of the root to access the surface layers of the soil where Pi availability is typically higher.

Plant hormones, in particular ethylene and auxin, can work as positive regulators of root hair development in tandem with environmental signals (Pitts *et al.*, 1998; Martín-Rejano *et al.*, 2011; Lee and Cho, 2013). Ethylene stimulates root hair development as it influences both hair density (specification and initiation) and length (elongation) (Masucci and Schiefelbein, 1994; Tanimoto *et al.*, 1995; Pitts *et al.*, 1998). In Arabidopsis, the ethylene response pathway is well characterized; ethylene is perceived by five receptors associated with the endoplasmic reticulum (Ji and Guo, 2013). In the absence of ethylene, the receptors activate CTR1 (CONSTITUTIVE TRIPLE1 RESPONSE1), which suppresses the function of its downstream target EIN2 (ETHYLENE INSENSTIVE2). When ethylene binds to the receptors, it inactivates CTR1, which causes the translocation of a C-terminal fragment of EIN2 (EIN2-C′) into the nucleus. EIN2-C′ then activates two key transcription factors EIN3 (ETHYLENE INSENSITIVE3) and EIL1 (ETHYLENE INSENSITIVE3-LIKE1), which then turn on the transcription of a chain of downstream target genes, including plant antimicrobial peptide *DEFENSIN* (*PDF1.2*; Penninckx *et al.*, 1998), which initiates a plethora of plant responses (Guo and Ecker, 2004; Li *et al.*, 2015; Song *et al.*, 2016). In addition, ethylene can affect primary root growth by regulating cell division and expansion as it induces cell division in the root meristem zone (MZ; Ortega-Martínez *et al.*, 2007), and inhibits cell expansion in the elongation zone (EZ; Le *et al.*, 2001). Dicotyledonous etiolated seedlings display a characteristic ‘triple response’ when treated with ethylene, manifested by the inhibition of root and hypocotyl growth, radial swelling of the hypocotyl, and exaggerated curvature of the apical hook (Guzmán and Ecker, 1990). In the past two to three decades, more than a dozen unique mutants have been identified with typical phenotypes; for example the ethylene-insensitive mutants *etr1-3* and *ein2-1* exhibit increased cell elongation, and the constitutive ethylene-response mutant *ctr1-1* has reduced cell elongation and increased root hair growth compared with wild-type plants (Stepanova and Ecker, 2000; Le *et al.*, 2001; Song *et al.*, 2016).

Plant cell walls are essential in determining the form of cells during plant growth (Keegstra, 2010). The root epidermis is an excellent tissue to study cell wall structure and function (Foreman and Dolan, 2001). Many genes impact on root epidermal cell specification and initiation, for example *TRANSPARENT TESTA GLABRA* (*TTG*), *GLABRA2* (*GL2*), *ROOT HAIR DEFECTIVE 6* (*RHD6*) (Galway *et al.*, 1994; Di Cristina *et al.*, 1996; Wada *et al.*, 1997; Lee and Schiefelbein, 1999; Bernhardt *et al.*, 2003; Menand *et al.*, 2007), but only a few genes are known to affect root hair tip growth (Won *et al.*, 2009). For example, the two root hair-specific *expansin* genes *AtEXPA7* and *AtEXPA18* promote hair initiation and elongation, indicating that expansins may affect the interactions between cellulose and xyloglucans for cell wall flexibility in root hair growth (Cho and Cosgrove, 2002; Lin *et al.*, 2011).

In the non-growing tubular portion of root hairs, organized cellulose microfibrils form thick and sturdy cell walls that prevent unwanted outgrowth, whereas at the growing tips, unordered cellulose microfibrils support thin and dynamic cell walls and provide the major strength to expanding cell walls (Sassen *et al.*, 1985; Somerville *et al.*, 2004; Galway, 2006; Taylor, 2008; Park *et al.*, 2011). Cellulose is synthesized by cellulose synthase (CesA) complexes, and CesA1, CesA3, and a CesA6-like protein are required for primary wall cellulose synthesis (Li *et al.*, 2013; Schneider *et al.*, 2016). Although mutations in *CesA1* (*rsw1-1*) or *CesA6* (*prc1-1*) affect root-hair development, tip growth is not abolished (Arioli *et al.*, 1998; Fagard *et al.*, 2000; Singh *et al.*, 2008). Accordingly, fluorescently labelled CesA proteins are not found at the apical plasma membrane region of growing root hairs (Park *et al.*, 2011). Instead, a fluorescently labelled cellulose synthase-like D3 (CSLD3) protein has been observed at this region, supporting a role of CSLD3 in root hair growth. Indeed, mutations in CSLD3, e.g. *kjk*, *csld3-1*, *rhd7-1*, and *rhd7-4*, abolish root-hair tip growth (Favery *et al.*, 2001; Wang *et al.*, 2001; Bernal *et al.*, 2008; Galway *et al.*, 2011). However, the function of CSLD3 in cell wall synthesis remains unclear and it has been variously suggested to be involved in the synthesis of cellulose, xylan, mannan, or homogalacturonan polysaccharides (Bernal *et al.*, 2007; Wang *et al.*, 2010; Park *et al.*, 2011; Verhertbruggen *et al.*, 2011; Yin *et al.*, 2011; Guo *et al.*, 2014).

Despite detailed genetic analyses of *AtCSLD3*-null mutants, much remains unknown about the functional context of CSLD3 during root-hair tip growth and plant growth, especially with regards to hormones and nutrients. In this study, we demonstrate that *CSLD3* acts downstream of the ethylene response and phosphate pathways in the control of root and root-hair elongation, and provide compelling evidence for how CSLD3 regulates general cell expansion via its impact on cellulose synthesis.

## Materials and methods

### Amplification of the *GhCSLD3* gene from cotton

To isolate the *CSLD3* gene in cotton (*Gossypium hirsutum* L.), the *Arabidopsis thaliana CSLD3* cDNA sequence (*At3g03050*) was compared with cotton databases (https://www.cottongen.org/) using BLASTN, and contig21384 was found to be the characteristic root of the target gene (Supplementary Fig. S1A at *JXB* online). To obtain the full-length gene, a segmented amplification method was used and primers were designed for contig21384 (see Supplementary Fig. S1B and Table S1). First, the middle fragment was amplified by the sense primer GhD33GSPL and the antisense primer GhD33GSPR. Second, the 5′- and 3′-end fragments were amplified by RACE-PCR. More specifically, we amplified the 5′-end fragment using outer primers (5′ RACE outer and HD35P1-out) and inner primers (5′ RACE inner and HD35P2-in) using a TaKaRa 5′- Full RACE Kit (Code: D315) and we used nested PCR to amplify the 3′-end fragment through double-PCR using outer primers (HD33P1-out and GhBC1-out) and inner primers (HD33P2-in and GhBC1-in). Third, the overlap-extension PCR method was applied to get the full-length sequence using the sense primer D3-FW2 and the antisense primer D3-RV combined with GhD33GSPR and GhD33GSPL, respectively. Finally, a 3815-bp fragment was amplified from young roots in cotton. The fragment consisted of a single 3366-bp ORF and was predicted to encode a protein of 1121 amino acid residues.

### Phylogenetic analysis

A phylogenetic tree was developed using the CLUSTALW program (http://www.ebi.ac.uk/clustalw/), choosing the Construct/Test Neighbor-Joining Tree (NJ) algorithm, and was visualized using the TREEVIEW software (http://taxonomy.zoology.gla.ac.uk/rod/treeview.html) for six AtCSLD proteins, five OsCSLD proteins, and the target protein GhCSLDx.

### Plant material and growth conditions

All Arabidopsis samples used in this study were of the Columbia ecotype (Col-0) background. Homozygous mutants of *csld3-1* (*AtCSLD3*) have been described previously (Wang *et al.*, 2001). The SALK T-DNA insertional lines (*SALK_059754* and *SALK_063966*) were obtained from the Arabidopsis Biological Resource Center (ABRC). Identification of T-DNA homozygous lines (*t-ein2* and *t-pdf1.2*) was done by using the three primers (LBb1.3+LP+RP) designed in T-DNA Primer Design (http://signal.salk.edu/tdnaprimers.2.html). For generation of RNAi (RNA interference) constructs of *AtCTR1* and *AtPDF1.2*, the RNAi sequences were generated based on the GeneSil website (http://www.genesil.com/siRNAdesign.asp). The corresponding 626- and 227-bp fragments were cloned into the middle vector PHANNIBAL and then connected to vector PART27. For generation of overexpression constructs, complete *AtCSLD3* and *GhCSLD3* coding regions driven by the *D35S* promoter were cloned into the binary vector pD1301s to generate the binary plasmids. Transgenic plants were generated by introduction of the plant expression constructs into *Agrobacterium tumefaciens* strain GV3101 and transformation was done by floral dipping (Zhang *et al.*, 2006). The overexpression constructs were transformed into the wild-type (WT; for overexpression), *csld3-1* (for complementation testing), and the *i-ctr1*, *t-ein2*, and *t-pdf1.2* mutants. Transformed plants were selected on plates containing kanamycin (50 mg ml^–1^) for RNAi lines and plates containing hygromycin (50 mg ml^–1^) for overexpression lines. More than 20 independent transformants were selected. All the primers are listed in Supplementary Table S3. The *i-ctr1 csld3-1* and *csld3-1 i-ctr1* double-mutants were identified from the F_2_ offspring of hybrids between the *i-ctr1* and *csld3-1* mutants by kanamycin screening, and we obtained homozygous double-mutants from the F_3_ offspring.

Arabidopsis seeds were surface-sterilized using 75% ethanol for 4 min and 10% sodium hypochloride with 0.01% Triton X-100 for 3 min, and were then washed in sterile water several times. Seeds were then imbibed at 4 °C in the dark in sterile water containing 0.1% agar for 3 d, and germinated on plates containing half Murashige and Skoog (MS) media (1% sucrose; pH 5.8) in 1% agar. Plates were incubated in a near-vertical position at 22 °C under 16 h light/8 h dark conditions (light-grown) for photomorphogenesis, or under 24 h dark conditions (dark-grown) for skotomorphogenesis. The seedlings were transplanted into soil after the second real leaf was visible.

### Ethylene precursor and phosphate starvation treatments

For the ethylene precursor treatment, seeds were sown directly on half-MS medium containing either 1 μM or 5 μM ACC (1-aminocyclopropane-1-carboxylic acid). After the seeds had been stratified at 4 °C for 3 d, they were placed horizontally in a growth room with a 16-h light/8-h dark cycle at 22 °C for 6 d. For the triple-response test, the growth conditions were 24-h dark at 22 °C for 4 d. For the phosphate starvation (P–) treatment, seeds were sown directly either on P– medium or P– medium containing 1 μM ACC: in the P– medium, the 1.25 mM KH_2_PO_4_ in the half-MS medium was replaced with 0.65 mM K_2_SO_4_, as described previously (Song *et al.*, 2016).

### Root hair, root, and hypocotyl phenotype analysis

Root-hair morphology was examined using 6-d-old light-grown (L6) seedlings. To measure the root-hair length, the differentiation zones (DZs) with root hairs were viewed under light microscopy using a Leica stereomicroscope (Leica S6 D, with Leica DFC295 digital camera). The length of root hairs in these images was measured using the ImageJ 1.32j software (https://imagej.nih.gov/ij/).

To observe root and hypocotyl growth, Arabidopsis seedlings were scanned using a HP Scanjet 8300 scanner at 600 dpi and then analysed using ImageJ 1.32j. Measurements were made of the L6 root length from the root tip to hypocotyl base, and of the 4-d-old dark-grown (D4) hypocotyl length of vertically grown seedlings from the hypocotyl base to the apical hook.

For images of epidermal cell patterns, D4 hypocotyls (2^nd^ to 6^th^ cells in the basal part) and L6 roots (the fully expanded cells in the EZ) were mounted in chloral hydrate and images were viewed using differential interference contrast (80i; Nikon, Japan). The EZ of the root is between the MZ (indicated by small and closely aligned cortical cells) and the DZ (indicated by the formation of root hairs). Epidermal cell lengths in recorded images (D4 hypocotyls and L6 roots) were quantified using Image J 1.32j, and epidermal cells of hypocotyls were visualized under confocal laser scanning microscopy (p58; Leica, Leica Microsystems, Nussloch, Germany) using D4 hypocotyls incubated in the dark for 10 min in a fresh solution of 15 mM (10 mg ml^–1^) propidium iodide (PI) (Naseer *et al.*, 2012). PI was excited at 488 nm, and fluorescence was detected at 600–700 nm.

Root meristem size (i.e. MZ) was determined as the distance between the quiescent center (QC) and the transition zone (TZ; the position of the first elongating cortical cell), and the number of cortical cells were counted in a file extending from the QC to the TZ (Beemster and Baskin, 1998). To count the number of cortical cells, L6 root MZs were mounted in chloral hydrate, and images were taken using differential interference contrast (80i; Nikon, Japan).

All the experiments were done using at least three biological replications and at least 20 seedlings were measured in each replicate. Least-significant difference (LSD) tests (*P*<0.01) were used for multiple comparisons.

### mRNA expression analyses

RNA extraction and RT-PCR analyses of cotton tissues were done using the method described by Wu and Liu (2004). *GhUBQ7* was used as the internal control. L6 Arabidopsis seedlings were germinated and grown on half-MS medium and were placed in liquid nitrogen at harvest. Total RNA was isolated from the collected tissues using Trizol reagent (Invitrogen, Carlsbad, CA, USA). The RNA concentration was determined using a NanoDrop ND-1000 spectrophotometer (Thermo Scientific) and about 10 μg RNA of each sample was transcribed into cDNA. First-strand cDNA was obtained using OligodT and M-MLV reverse transcriptase (Promega, Madison, WI, USA). Q-PCR amplification was carried out on a Bio-Rad MyCycler thermal cycler with SYBER Premix ExTaq (TakaRa, Tokyo, Japan) according to the manufacturer’s instructions, and *AtGAPDH* was used as the internal control. The PCR thermal cycle conditions were as follows: one cycle of 95 °C for 2 min, followed by 45 cycles of 95 °C for 15 s, 58 °C for 15 s, and 72 °C for 25 s. The expression value of *AtGAPDH* was defined as 100, and the expression levels of genes were normalized accordingly. All of the primers used in these assays are listed in Supplementary Table S2, and the assays were carried out for three biological replicates. LSD tests (*P*<0.01) were used for multiple comparisons.

### Immunolocalization of glycan epitopes

L6 roots and the inflorescence 1^st^ internode from 7-week-old plants were embedded with 4% agar and then cut into 60-μm sections using a microtome (VT1000S, Leica). For immunolabelling, transverse sections were incubated in 3% (w/v) milk protein in 1× PBS (MP/PBS) for 1 h to block non-specific binding. Sections were then incubated for 1 h with the following monoclonal antibodies diluted by 1:5 in MP/PBS: CCRC-M93, CCRC-M38, CCRC-M35, and CCRC-M149 (http://glycomics.ccrc.uga.edu/wall2/antibodies/antibodyHome.html), which bind to xyloglucan, de-esterified homogalacturonan, rhamnogalacturonan I, and xylan, respectively (Pattathil *et al.*, 2010; DeMartini *et al.*, 2011). After washing with PBS, sections were incubated with anti-mouse-IgG linked to fluorescein isothiocyanate (FITC; Sigma), and diluted 1:1000 in 1× PBS for 1 h. Counterstaining was performed with Calcofluor White M2R fluorochrome (fluorescent brightener 28; Sigma; 0.25 μg ml^–1^ in dH_2_O). Immunofluorescence was observed with an epifluorescence microscope (Olympus BX-61, with Retiga-4000DC digital camera) equipped with the following filter sets: 330~385/450 nm (excitation/emission; ex/em) for visualizing cell walls stained with Calcofluor White (Haigler *et al.*, 1980), and 460~490/520 nm (ex/em) for green emission of the FITC fluorochrome.

### Crystalline cellulose extraction

L6 roots were detached, freeze-dried, and ground into powder. Powder samples (40 mesh, 0.1~1.0 g) from both L6 roots and stems from 7-week-old plants were suspended in 5.0 ml acetic acid–nitric acid–water (8:1:2, v/v/v) and heated for 1 h in a boiling water bath with stirring every 10 min. After centrifugation, the pellets were washed several times with 5.0 ml water, and the resulting pellets were defined as the crystalline cellulose samples. The extractions were carried out with three biological replicates. LSD tests (*P*<0.01) were used for multiple comparisons.

### Determination of neutral sugars in the total wall polysaccharides by GC-MS

Powder (40 mesh, 0.1~1.0 g) samples from both L6 roots and stems of 7-week-old plants were washed twice with 5.0 ml phosphate buffer and twice with 5.0 ml distilled water. The remaining pellets were stirred with 5.0 ml chloroform–methanol (1:1, v/v) for 1 h at 40 °C and washed twice with 5.0 ml methanol, followed by 5.0 ml acetone. The pellets were then washed once with 5.0 ml distilled water. A 5.0-ml aliquot of DMSO-water (9:1, v/v) was added to the remaining pellets and they were vortexed for 3 min and then rocked gently on a shaker overnight. After centrifugation, the pellets were washed twice with 5.0 ml DMSO-water, and then with 5.0 ml distilled water three times. The resulting pellets were defined as the total wall polysaccharides. Then, the pellets were dissolved in 2 M trifluoroacetic acid (TFA) and the neutral sugars were determined by GC-MS as described previously (Xu *et al.*, 2012). Three biological replications were performed.

## Results

### Functional characterization of a cotton *CSLD3* gene in Arabidopsis

Since there has been little research on *CSLD* genes in cotton, we first isolated a *CSLD*-related gene from young cotton roots through RACE-PCR amplification (see Supplementary Fig. S1). The putative *GhCSLD*3 gene consisted of a 3366-bp ORF encoding a protein of 1121 amino acid residues, showing 78% and 84% identity with the Arabidopsis *AtCSLD3* gene and protein, respectively (Supplementary Fig. S1C). Like other CSLDs, the GhCSLD3 protein also contained a ‘D_D_D_QxxRW’ motif, and appeared in close proximity to AtCSLD2 and AtCSLD3 in a phylogenetic tree (Supplementary Fig. S1D).

**Fig. 1.**
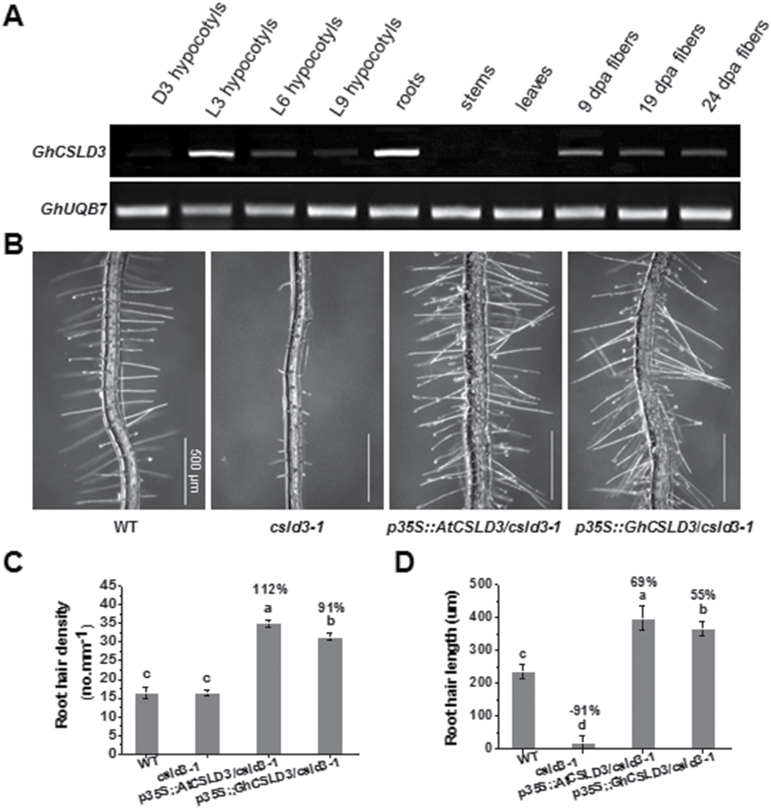
Genetic complementation test of the Arabidopsis root hairless *csld3-1* mutant by *AtCSLD3* and *GhCSLD3*. (A) Expression of *GhCSLD3* in different organs in *Gossypium hirsutum* assessed by RT-PCR. *GhUBQ7* was used as the internal control. D indicates seeds germinated and grown on half-MS media under dark (24 h dark) conditions, L indicates light (16 h light:8 h dark) conditions; the number of days of growth is indicated. dpa, days post-anthesis. (B) Root hair patterns of Arabidopsis wild-type (WT), *csld3-1*, and complementation lines for 6-d-old light-grown (L6) seedlings on half-MS media. (C, D) Quantitative analyses of root-hair density (C) and length (D) of seedlings as shown in (B). Data are means ±SD (three biological replicates), *n*≥20 seedlings were measured in each replicate. LSD (least-significant difference) tests were used for multiple comparisons. Different letters above bars indicate that the means differ according to ANOVA and LSD tests (*P*<0.01). The percentage values (%) were calculated by subtraction from the WT value and divided by the WT. Scale bars indicate 500 μm.

Based on RT-PCR analyses, we found that *GhCSLD3* was mainly expressed in cotton roots, in hypocotyls of 3-d-old light-grown (L3) plants, and in fibers at 9 d post-anthesis (dpa) (Fig. 1A). By comparison, *AtCSLD3* is also highly expressed in roots, and mutations in the Arabidopsis gene cause defects in root-hair tip growth (Favery *et al.*, 2001; Wang *et al.*, 2001; Galway *et al.*, 2011). Over-expression of *GhCSLD3* or *AtCSLD3* under the control of a 35S promoter in the Arabidopsis *csld3-1* mutant fully restored root-hair (Fig. 1B) and root growth (Supplementary Fig. S2), indicating that GhCSLD3 could functionally rescue the *csld3-1* mutant defects. Notably, both the *csld3-1* lines overexpressing *GhCSLD3* and *AtCSLD3* exhibited significantly increased root-hair length and density as compared to the wild-type (WT) (Fig. 1C, D). These data indicate that CSLD3 promotes root hair growth.

### Overexpression of *AtCSLD3* or *GhCSLD3* leads to enhanced cell elongation and root-hair growth

**Fig. 2.**
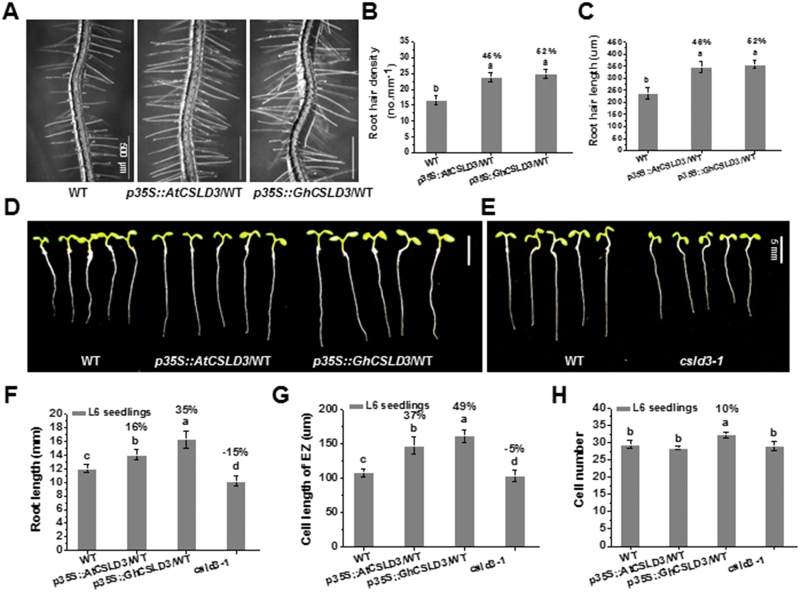
Phenotypes of the wild-type (WT), *csld3-1*, and *CSLD3*-overexpressing seedlings. (A) Root hair patterns of the WT, *AtCSLD3*-, and *GhCSLD3*-overexpressing seedlings on half-MS media. Seedlings were grown under light conditions and were 6-d old (L6). (B, C) Quantitative analyses of root-hair density (B) and length (C) for seedlings shown in (A). (D, E) WT, *AtCSLD3*-, and *GhCSLD3*-overexpressing L6 seedlings grown on half-MS media. (F) Measurements of root lengths of seedlings shown in (D, E). (G, H) Quantitative analyses of (G) cell lengths (the fully expanded cells) in the root elongation zone (EZ) and (H) cell numbers in the root meristem zone (MZ) of seedlings shown in (D, E). Data are means ±SD (three biological replicates); the number of seedlings measured in each replicate were: *n*≥20 in (B, C), *n*≥50 in (F), *n*≥30 in (G, H). Least-significant difference (LSD) tests were used for multiple comparisons. Different letters above bars indicate that the means differ according to ANOVA and LSD tests (*P*<0.01). The percentage values (%) were calculated by subtraction from the WT value and divided by the WT. Scale bars indicate 500 μm in (A), 5 mm in (D, E). (This figure is available in colour at *JXB* online.)

To corroborate the effects of AtCSLD3 and GhCSLD3 on root-hair growth, we also transformed the WT with the *AtCSLD3*- and *GhCSLD3*-overexpression constructs (Fig. 2A; Supplementary Fig. S4A, B and Table S2). Compared with the WT, both the *p35S::AtCSLD3/*WT and *p35S::GhCSLD3/*WT transgenic seedlings exhibited increased root-hair length and density of up to 50% (Fig. 2A–C). Interestingly, the transgenic lines also displayed longer roots, whereas the *clsd3-1* mutant exhibited shorter roots (Fig. 2D–F). Arabidopsis primary roots contain three distinct regions, namely the meristematic zone (MZ), the elongation zone (EZ), and the differentiation zone (DZ) (Somssich *et al.*, 2016). To assess in what region of the root the improved growth of the transgenic lines occurred, we measured the cell number of the MZ and the cell length of the EZ (for the fully expanded cells) in the different lines. Both the *p35S::AtCSLD3/*WT and *p35S::GhCSLD3/*WT transgenic lines exhibited much increased cell lengths (+37% and +49%, respectively) compared to the WT (Fig. 2G). By contrast, *csld3-1* displayed decreased cell lengths (–5%) compared to the WT. Interestingly, *p35S::GhCSLD3/*WT seedlings also showed significantly increased cell numbers in the MZ (Fig. 2H). Taken together, these data indicate that CSLD3 positively affects root-hair growth, cell elongation, and cell division during root growth.

### Ethylene-response mutants mimic CSLD3-related root growth phenotypes

**Fig. 3.**
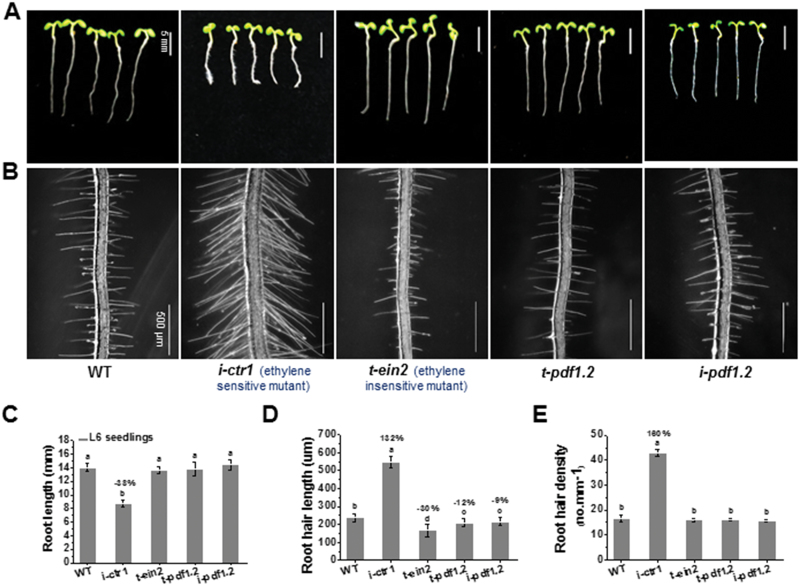
Analyses of growth characteristic of Arabidopsis mutants related to ethylene-response pathways. (A, B) Growth characteristics of L6 seedlings (i.e. 6-d-old, grown under light conditions) in the wild-type (WT), *i-ctr1*, *t-ein2*, *t-pdf1.2*, and *i-pdf1.2*. (C–E) Quantitative analyses of the seedlings shown in (A, B). Data are means ±SD (three biological replicates), *n*≥30 seedlings were measured in each replicate. Least-significant difference (LSD) tests were used for multiple comparisons. Different letters above bars indicate that the means differ according to ANOVA and LSD tests (*P*<0.01). The percentage values (%) were calculated by subtraction from the WT value and divided by the WT. Scale bars indicate 5 mm in (A), 500 μm in (B). (This figure is available in colour at *JXB* online.)

Because ethylene induces root-hair growth and inhibits primary root elongation (Le *et al.*, 2001; Ortega-Martínez *et al.*, 2007), we aimed to investigate potential links between CSLD3 function and ethylene in root development. We therefore obtained T-DNA insertion mutants and generated RNAi lines of some representative genes in the ethylene response pathway (*AtCTR1*, *AtEIN2*, *AtPDF1.2*; T-DNA insertion mutants *t-ein2*, *t-pdf1.2* and RNAi lines *i-ctr1*, *i-pdf1.2*; see Supplementary Fig. S3 and Table S3). Mutants and RNAi lines were confirmed by Q-PCR analyses (Supplementary Fig. S4C and Table S2). As compared to the WT, the *i-ctr1* lines exhibited remarkably reduced primary root growth and increases in root-hair length and density; by contrast, the other mutants/lines (*t-ein2*, *t-pdf1.2*, and *i-pdf1.2*) showed a reduction in root-hair length (Fig. 3), consistent with previous reports on ethylene-sensitive and -insensitive mutants (Kieber *et al.*, 1993; Alonso *et al.*, 1999).

### Lesions in CSLD3 abolish ethylene-induced root-hair tip growth

**Fig. 4.**
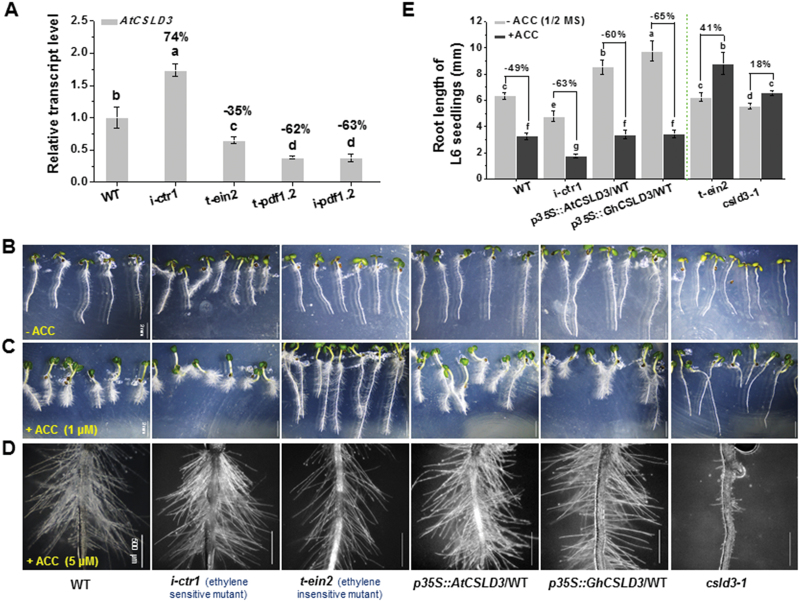
Comparison of ethylene responses among the wild-type (WT), *CSLD3*-overexpressing lines, *csld3*, *i-ctr1*, and *t-ein2* mutants. (A) Gene expression analysis of *AtCSLD3* in the WT and ethylene-response mutant seedlings grown on half-MS media. Seedlings were grown under light conditions and were 6-d old (L6). Data are means ±SD (three biological replicates); the results of least-significant difference (LSD) tests are indicated (*P*<0.01). The percentage values (%) were calculated by subtraction from the WT value and divided by WT. (B–D) Morphological phenotypes of L6 seedlings grown in the absence or presence of ACC. Seedlings were grown on half-MS media alone (B), with addition of 1 μM ACC (C), or with addition of 5 μM ACC (D). (E) Quantitative analyses of root lengths of seedlings as shown in (B, C). Data are means ±SD (three biological replicates), *n*≥30 seedlings were measured in each replicate. LSD tests were used for multiple comparisons. Different letters above bars indicate that the means differ according to ANOVA and LSD tests (*P*<0.01). Scale bars indicate 2 mm in (B, C); 500 μm in (D). (This figure is available in colour at *JXB* online.)

To understand the potential relationship between CSLD3 and ethylene, we assessed the *AtCSLD3* expression levels in the ethylene response-pathway mutants. Interestingly, expression of *AtCSLD3* was significantly increased in *i-ctr1* and decreased in *t-ein2*, *t-pdf1.2*, and *i-pdf1.2* (Fig. 4A). These data suggested that the expression of *AtCSLD3* may be regulated by the ethylene-response pathway. To first assess how the *CSLD3* transgenic and mutant lines were affected by ethylene, we grew all the lines on ACC, an ethylene precursor (Masucci and Schiefelbein, 1994, 1996). When grown on media supplemented with 1 μM or 5μM ACC, the *CSLD3* overexpression and *csld3-1* complemented lines, the ethylene-response mutants, and the WT all exhibited a dramatic increase in root-hair density and length (Fig. 4B–D; Supplementary Fig. S5A–C). Notably, neither the 1 μM nor the 5 μM ACC treatments induced root-hair tip growth in the *csld3-1* mutant (Fig. 4B, C). Indeed, the *csld3-1* mutants grown on 5 μM ACC showed a maximum root-hair length of 35 μm (WT: 236.58 ± 23.27 μm on half-MS; Fig. 4D; Supplementary Fig. S6B).

We next looked at primary root growth and, interestingly, root length displayed different responses depending on genotype. Compared with the WT and the two *CSLD3* complementary lines (decreased by 49%, 46%, and 51%, respectively, on 1 μM ACC), the *CSLD3* overexpressing roots displayed increased sensitivity to ACC (decreased by 60% and 65%), which was also the case for the *i-ctr1* mutant seedlings (decreased by 63%; Fig. 4E; Supplementary Fig. S5G). However, the *csld3-1* mutant was insensitive to ACC, similar to the ethylene-insensitive and non-responsive mutants *t-ein2*, *t-pdf1.2*, and *i-pdf1.2* (Fig. 4E; Supplementary Figs. S5G, S6A). Hence, while the root elongation of the *CSLD3*-overexpressing plants was more sensitive to ethylene, plants lacking *CSLD3* were insensitive to ethylene; both in terms of root-hair tip growth and root elongation, which correlated well with ethylene-related signaling processes.

**Fig. 5.**
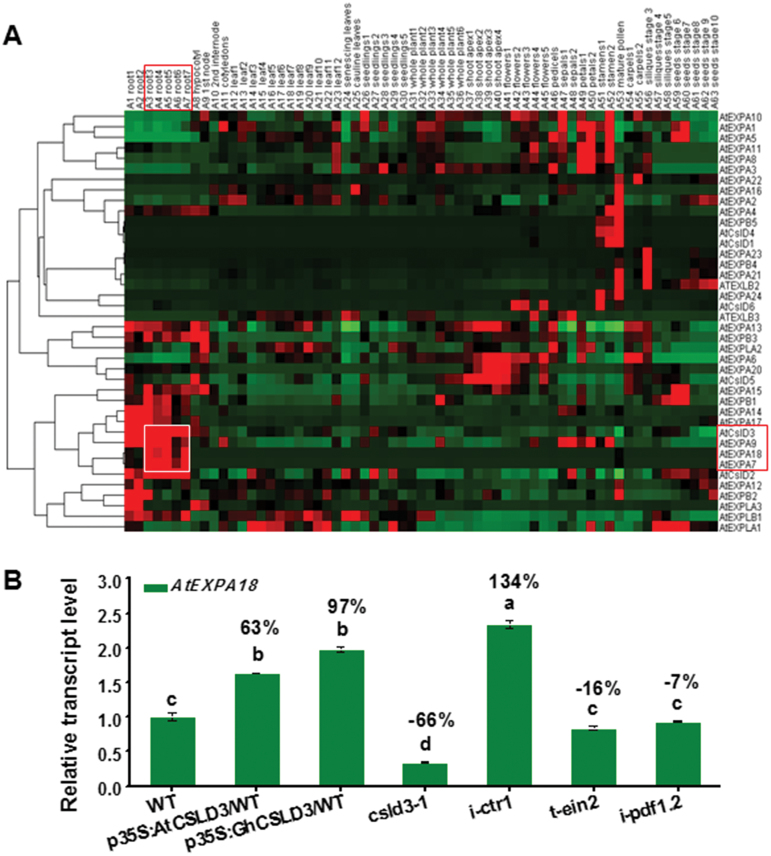
Expression patterns of *AtCSLD3* and *AtEXPA18*. (A) Expression profile of co-expression of *expansins* and the *CSLD* gene family in Arabidopsis. (B) *AtEXPA18* expression levels in the wild-type (WT), *AtCSLD3*-, and *GhCSLD3*-overexpressing lines, and the mutants *csld3-1*, *i-ctr1*, *t-ein2*, *t-pdf1.2*, and *i-pdf1.2* seedlings grown on half-MS. Seedlings were grown under light conditions and were 6-d old (L6). Data are means ±SD (three biological replicates). Least-significant difference (LSD) tests wwereas used for multiple comparisons. Different letters above bars indicated that the means differ according to ANOVA and LSD tests (*P*<0.01). The percentage values (%) were calculated by subtraction from the WT value and divided by the WT.

In addition, it has been reported that two root hair-specific expansin genes (encoding cell wall proteins), *AtEXPA7* and *AtEXPA18*, may also act downstream of hormones (e.g. ethylene and auxin) to affect root hair initiation and elongation (Cho and Cosgrove, 2002; Lin *et al.*, 2011). Here, we found *AtEXPA18*, and its family genes *AtEXPA7* and *AtEXPA9*, were highly co-expressed with *AtCSLD3* in the root tissues (Fig. 5A). Indeed, the expression of *AtEXPA18* increased in the *CSLD3*-overexpressing lines and decreased in the *csld3-1* mutant (Fig. 5B). However, while *AtEXPA18* increased in the *i-ctr1* mutant, it showed no significant changes in the *t-ein2* and *i-pdf1.2* mutants (Fig. 5B), which was somewhat differ to that of the expression of *AtCSLD3* in young seedlings (Fig. 4A), suggesting that *AtEXPA18* and *AtCSLD3* may somehow work together in root-hair growth but perhaps through different pathways.

### CSLD3 acts downstream of the phosphate starvation and enhanced-ethylene signaling pathways in root-hair and root growth

**Fig. 6.**
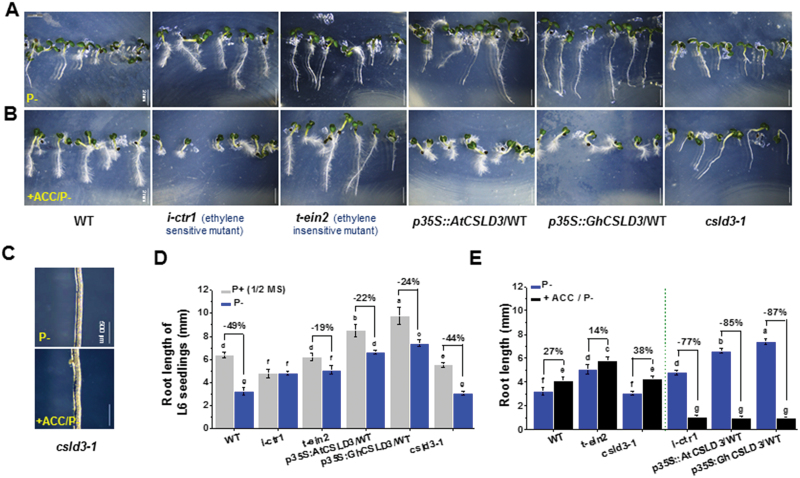
Increased ethylene sensitivity of root elongation under phosphate starvation (P–). (A, B) Morphological phenotypes of 6-d-old seedlings grown under light conditions (L6). Seedlings grown on P– media (A) or P– co-supplied with 1 μM ACC (B). (C) A close-up view of the root hair patterns in the *csld3-1* mutant. (D, E) Quantitative analyses of root lengths of seedlings as shown in (A, B). Data are means ±SD (three biological replicates), *n*≥30 seedlings were measured in each replicate. Least-significant difference (LSD) tests were used for multiple comparisons. Different letters above bars indicate that the means differ according to ANOVA and LSD tests (*P*<0.01). Scale bars indicate 2 mm in (A, B); 500 μm in (C). (This figure is available in colour at *JXB* online.)

Phosphate starvation (P–) inhibits primary root growth but increases root-hair elongation (López-Arredondo *et al.*, 2014), and we therefore hypothesized that CSLD3 might also work in the context of phosphate perception and signaling. To test this, we grew Arabidopsis seedlings from the different genotypes on P– media. This treatment led to increased root-hair tip growth of the *CSLD3*-overexpressing and complemented lines, all the ethylene-response mutants, and the WT, but it failed to induce root-hair tip growth in the *csld3-1* mutant ((Fig. 6A, C; Supplementary Fig. S5D, E). Because ethylene plays an important role in P starvation-induced root-hair development (Song *et al.*, 2016), we grew Arabidopsis seedlings on P– media containing 1 μM ACC and found that, indeed, the P– treatment did cause a further enhancement of root-hair growth in all the genotypes with the exception of *csld3-1* (Fig. 6B, C; Supplementary Fig. S5F). These data suggest that CSLD3 works downstream of the ethylene and phosphate signaling pathways during root-hair growth in Arabidopsis.

We next investigated how phosphate interacted with ethylene and CSLD3 during root growth. Similar to the root-hair growth estimates, we first assessed the root growth on P– media. Here, the *CSLD3*-overexpressing lines and the ethylene-response mutants showed similar growth responses, which were less affected compared to the WT, the *CSLD3* complemented lines, and the *csld3-1* mutant plants (Fig. 6D; Supplementary Fig. S5H). We also grew the lines on P– media supplemented with ACC and measured the effects on root growth. Interestingly, here the WT, *CSLD3* complemented, *t-ein2* and the *csld3-1* mutant lines behaved very similarly to each other, with very minor growth differences as compared to roots grown with the P– treatment (Fig. 6E; Supplementary Fig. S5I). In contrast, the root growth of the *CSLD3*-overexpressing lines and *i-ctr1*, *t-pdf1.2*, and *i-pdf1.2* were strongly inhibited by the addition of 1 μM ACC. These data suggest that although CSLD3 is important for how roots grow in response to ethylene and phosphate, clearly PDF1.2 contributes another intersection point in this path.

**Fig. 7.**
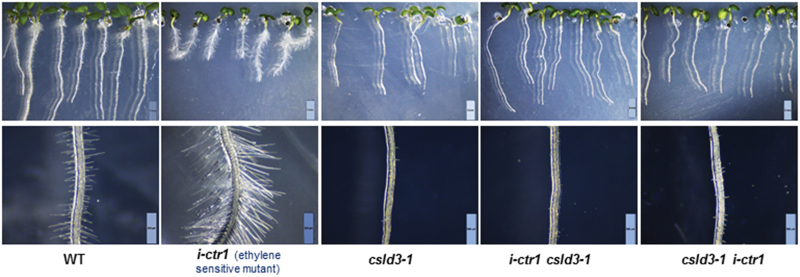
The homozygous *i-ctr1 csld3-1* and *csld3-1 i-ctr1* double-mutants are hairless like the *csld3-1* parent. Morphological phenotypes of 9-d-old light-grown (L9) seedlings grown on half-MS media. Scale bars indicate 2 mm (top); 500 μm (bottom). (This figure is available in colour at *JXB* online.)

**Fig. 8.**
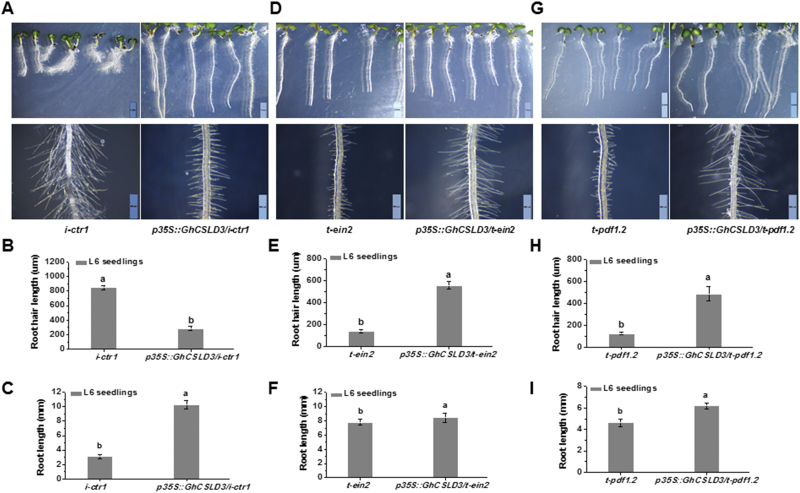
Overexpression of *GhCSLD3* in the *i-ctr1*, *t-ein2*, and *t-pdf1.2* mutants. (A, D, G) L6 seedlings (i.e. 6-d-old grown under light conditions) grown on half-MS media. Scale bars indicate 2 mm (top) and 500 μm (bottom). (B–I) Quantitative analyses of root-hair length (B, E, H) and root length (C, F, I) for the seedlings shown in (A, D, G). Data are means ±SD (three biological replicates); the number of seedlings measured in each replicate were: *n*≥20 in (B, E, H) and *n*≥50 in (C, F, I). Least-significant difference (LSD) tests were used for multiple comparisons. Different letters above bars indicate that the means differ according to ANOVA and LSD tests (*P*<0.01). (This figure is available in colour at *JXB* online.)

To further explore the association between the ethylene-response pathway genes and CSLD3, we constructed *i-ctr1 csld3-1* and *csld3-1 i-ctr1* double-mutants. Like *csld3-1*, the double-mutants did not produce root hairs (Fig. 7), suggesting that *CSLD3* acts downstream of *CTR1* in the control of root-hair tip growth. Consistent with this, overexpression of *CSLD3* in the *i-ctr1* mutant displayed markedly shorter root-hair length compared to the *i-ctr1* mutant, leading to a similar phenotype to the WT (Fig. 8A, B). By contrast, overexpression of *GhCSLD3* in the *t-ein2* and *t-pdf1.2* mutants led to markedly longer root-hair length (Fig. 8D, E, G, H). Moreover, all the transgenic lines showed longer root length (Fig. 8C, F, I), similar to that of the *CSLD3*-overexpressing lines (Fig. 2D, F). These data further support the notion that CTR1, EIN2, PDF1.2, and CSLD3 act in the same genetic pathway in the control of root and root hair elongation.

### CSLD3 has positive effects on hypocotyl elongation independently of the ethylene response pathway

**Fig. 9.**
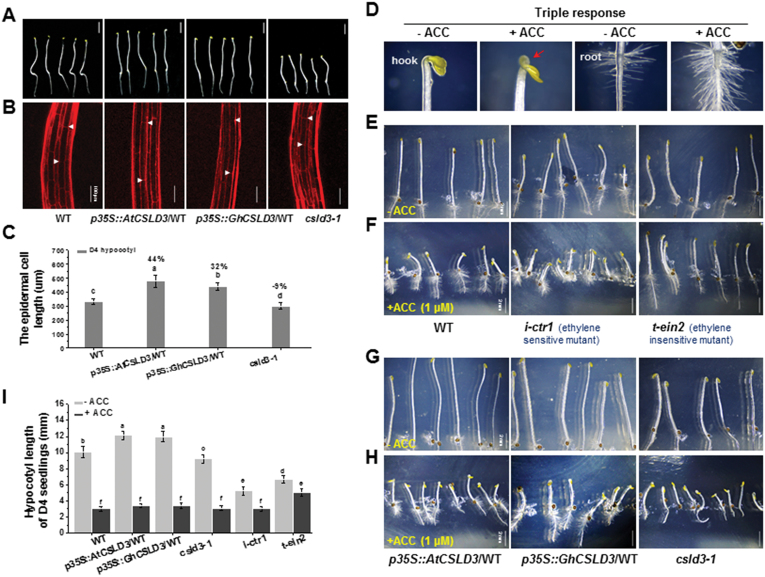
Triple response to ethylene among the wild-type (WT), *CSLD3*-overexpressing lines, and the *csld3*, *i-ctr1*, and *t-ein2* mutants. (A) Dark-grown 4-d-old (D4) seedlings grown on half-MS media. (B) Confocal laser scanning microscopy images of the basal region of the hypocotyl (2^nd^ to 6th cells) showing the longest epidermal cells (indicated by arrowheads) of the seedlings shown in (A). (C) Quantitative analyses of the longest epidermal cell lengths in the hypocotyls shown in (B). The percentage values (%) were calculated by subtraction from the WT value and divided by the WT. (D) Sample images of the growth characteristics of the ‘tripe response’ to ethylene. (E–H) Morphological phenotypes of D4 seedlings in the absence or presence of 1 μM ACC For the WT, *i-ctr1*, and *t-ein2* mutants (E, (F), and the *AtCSLD3*- and *GhCSLD3*-overexpressing lines, and the *csld3-1* mutant (G, H). (I) Quantitative analyses of the hypocotyl lengths in the seedlings shown in (E–H). Data are means ±SD (three biological replicates), *n*≥30 seedlings were measured in each replicate. Least-significant difference (LSD) tests were used for multiple comparisons. Different letters above bars indicate that the means differ according to ANOVA and LSD tests (*P*<0.01). Scale bars indicate 5 mm in (A); 100 μm in (B); 2 mm in (E–H). (This figure is available in colour at *JXB* online.)

To investigate whether CSLD3 affected cell elongation, we also examined etiolated hypocotyl growth. Compared with the WT, *CSLD3*-overexpressing seedlings exhibited increased hypocotyl lengths, whereas the *csld3-1* mutant had reduced lengths (Fig. 9A). Based on measurements of cells at the base of the hypocotyls (2^nd^ to 6^th^ cells), the *CSLD3*-overexpressing seedlings had much longer cells than the WT, and the *csld3-1* seedlings showed significantly shorter cells (Fig. 9B, C). Because the number of epidermal cells in a single, vertical cell file (parallel to the direction of growth) is genetically fixed at approximately 20 in hypocotyl tissues of Arabidopsis (Gendreau *et al.*, 1997), the data indicate that *CSLD3* positively affects cell elongation in Arabidopsis hypocotyls.

To again put this in the context of ethylene signaling, we grew etiolated Arabidopsis seedlings on media supplemented with 1 μM ACC. All seedlings exhibited the characteristic ‘triple response’ with exaggerated apical hook curvature, and reduced hypocotyl growth (Fig. 9D–H; Guzmán and Ecker, 1990; Song *et al.*, 2016), with the notable exception of the *t-ein2* mutants. Here, the *csld3-1* mutant exhibited clear hypocotyl growth inhibition in response to ACC, similar to what we observed in the *CSLD3*-overexpressing transgenic lines, the WT, and the *i-ctr1* mutants (Fig. 9I). These data suggest that, in contrast to root-hair tip and primary root growth, the positive impact of CSLD3 on hypocotyl elongation is probably independent of the ethylene-response pathway, or at least works in parallel with it.

### Changes in *CSLD3* expression affect cellulose synthesis

**Fig. 10.**
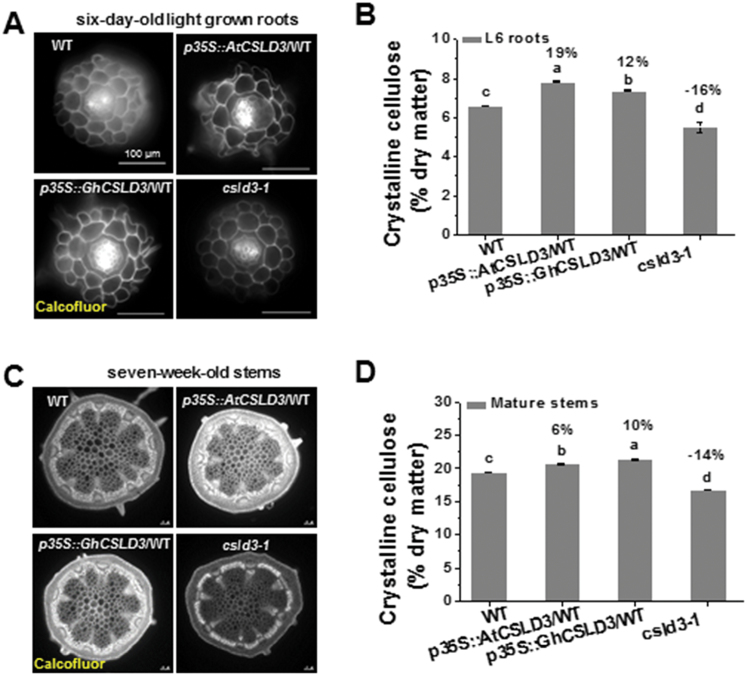
Analyses of cellulose production in roots of 6-d-old light-grown (L6) seedlings and 7-week-old stems. (A, C) Calcofluor staining for β-glucan. Transverse section of roots (A) and stems (C). (B, D) Detection of crystalline cellulose contents for roots (B) and stems (D). WT, wild-type. Data are means ±SD (three biological replicates). Least-significant difference (LSD) tests were used for multiple comparisons. Different letters above bars indicate that the means differ according to ANOVA and LSD tests (*P*<0.01). The percentage values (%) were calculated by subtraction from the WT value and divided by the WT. Scale bars indicate 100 μm in (A, C).

As CSLDs are reportedly associated with the biosynthesis of wall polysaccharides (xylan, pectin, and cellulose) (Bernal *et al.*, 2007; Park *et al.*, 2011; Verhertbruggen *et al.*, 2011; Yin *et al.*, 2011; Qi *et al.*, 2013), we examined how *CSLD3* impacted on the cell wall composition of L6 roots and 7-week-old stems. Using Calcofluor staining for β-glucans, we observed stronger fluorescence signals in the *CSLD3*-overexpressing lines than in the WT in the young roots (Fig. 10A). Chemical analyses revealed that the *CSLD3*-overexpressing lines had significantly increased crystalline cellulose (12–19% higher than the WT) (Fig. 10B). In contrast, the *csld3-1* mutant exhibited both relatively weaker Calcofluor staining, and reduced crystalline cellulose content (16% lower than the WT) (Fig. 10A, B). Using glycan antibodies for immunolabelling wall polymers *in situ*, we observed similar patterns and intensities for antibodies against xyloglucan, de-esterified homogalacturonan, and rhamnogalacturonan I in the WT, the *CSLD3*-overexpressing lines, and the *csld3-1* mutant (Supplementary Fig. S7A). Moreover, based on GC-MS analyses of neutral sugars within the total wall polysaccharides, both the *CSLD3*-overexpressing lines and the *csld3-1* mutant showed a significant increase in rhamnose and decreases in arabinose and mannose compared to the WT (Supplementary Fig. S7B); however, these changes were more difficult to discern than the changes in cellulose.

In addition, we found that the *CSLD3*-overexpressing lines also had higher crystalline cellulose levels (6–10%) than the WT in 7-week-old stems, whereas the *csld3-1* mutant showed reduced crystalline cellulose (14% lower than the WT) (Fig. 10C, D). Similar to seedling tissues, we did not find any differences in immunolabeling patterns in stems using xylan and de-esterified homogalacturonan antibodies (Supplementary Fig. S7C), and only slight changes in the neutral sugars within the total wall polysaccharides (Supplementary Fig. S7D). These data indicate that CSLD3 positively affects cellulose synthesis, with some moderate effects on other cell wall polymers.

## Discussion

**Fig. 11.**
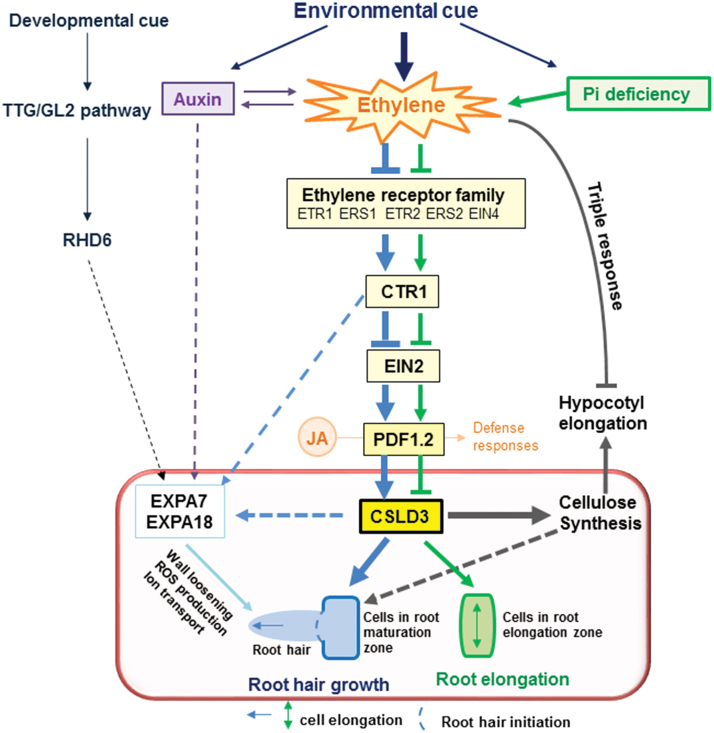
A model that illustrates how CSLD3 acts downstream of the ethylene-response pathway in root hairs and during cell elongation in Arabidopsis. CSLD3 controls root-hair growth and root elongation, probably through cellulose biosynthesis and cell wall formation. The model highlights that CSLD3-mediated root elongation is sensitive to ethylene treatment, in particular under phosphate starvation, but that CSLD3-mediated hypocotyl elongation proceeds largely independently of ethylene signaling. The model also assumes a co-ordinated effort between *CSLD3* and root hair-specific *expansin* genes. Arrows indicate positive regulation; lines ending in bars indicate negative action; solid lines indicate that the effects are experimentally supported; dashed lines indicate hypothetical links. (This figure is available in colour at *JXB* online.)

Root-hair development is influenced by many factors, such as developmental regulators, hormones, and the environment (Grierson and Schiefelbein, 2002). Ethylene has positive effects on both root-hair initiation and elongation (Tanimoto *et al.*, 1995; Pitts *et al.*, 1998). Although many mutants of key genes in the ethylene-signaling pathway (e.g. *etr1* and *ein2*) affect root-hair growth (Masucci and Schiefelbein, 1996), none of them display complete inhibition of root-hair tip growth, indicating the possible existence of some predominant downstream genes that control tip growth. Moreover, little has been reported on how genes associated with cell wall synthesis and re-modelling function in root-hair development during ethylene signaling, which is surprising as root-hair growth is dependent on rapid alteration of the cell wall. Nevertheless, two root hair-specific *expansin* genes (*AtEXPA7* and *AtEXPA18*; Cho and Cosgrove, 2002; Lin *et al.*, 2011) are known to act downstream of the ethylene-signaling pathway and to impact on root-hair elongation. In this study, through use of the *i-ctr1 csld3-1* double-mutant, we report that CSLD3 acts downstream of the ethylene-response pathway to control root and root-hair elongation (Fig. 7). Overexpression of *GhCSLD3* restored the root-hair defects in *csld3-1* (Fig. 1), which is consistent with overexpression of *PdCSLD5* and *PdCSLD6* (poplar orthologs to *AtCSLD3*) in the *atcsld3* mutant reported previously (Qi *et al.*, 2013). In addition, overexpression of either *AtCSLD3* or *GhCSLD3* increased the sensitivity of root and root-hair elongation to ethylene (Figs 4, 6), suggesting a role of CSLD3 in the ethylene pathway. Notably, we found similar, but also distinct, phenotypes between root hair-specific *expansin* and *CSLD3* mutants, suggesting that those two genes may work together in root-hair growth, but perhaps via involvement in different pathways, as shown in the proposed model presented in Fig. 11. Hence, our results provide insights into root-hair growth that should inform future studies.

Previous studies have revealed ethylene-mediated root-hair development under phosphate starvation (Song *et al.*, 2016), but little has been reported on root elongation. In this study, we showed that cell wall changes via CSLD3 influenced how ethylene and phosphate starvation affected root elongation. In particular, when treated with ethylene, the *CSLD3*-overexpressing lines and ethylene-sensitive mutant *i-ctr1* exhibited reduced root lengths under phosphate starvation (+ACC/P–), which was in contrast to the WT, *csld3-1*, and *t-ein2* mutants that were insensitive, or less sensitive, to ethylene (Figs 4E, 6E). Here, the *t-pdf1.2* and *i-pdf1.2* mutants acted in accordance with the *i-ctr1* rather than the *t-ein2* mutant (Supplementary Fig. S5I), perhaps because *AtPDF1.2* is also involved in other signaling pathways (e.g. jasmonate; Penninckx *et al.*, 1998).

Mutant alleles (*kjk/csld3-1/rhd*) of *AtCSLD3* exhibit inhibited root-hair tip growth (Favery *et al.*, 2001; Wang *et al.*, 2001; Galway *et al.*, 2011), but CSLD3 is also involved in other processes such as female gametophyte development and vegetative growth (Yin *et al.*, 2011; Yoo *et al.*, 2012). In particular, *CSLD2*, *CSLD3*, and *CSLD5* play important roles in various vegetative tissues during plant growth and development in Arabidopsis (Bernal *et al.*, 2008; Yin *et al.*, 2011; Yoo *et al.*, 2012). For instance, the *csld2csld5* and *csld3csld5* double-mutants show disturbed cell division and cell elongation in many tissues and organs. In addition, other studies have reported that *AtCSLD5* and its orthologous genes in rice and maize play a role in cell division during leaf development (Hunter *et al.*, 2012; Yoshikawa *et al.*, 2013; Gu *et al.*, 2016). In this study, we provided further evidence for how *CSLD3* functions in root and hypocotyl elongation. *CSLD3* promoted hypocotyl elongation in an ethylene-independent manner (Fig. 9), indicating that the role of CSLD3 in hypocotyl elongation is different from that in the roots. Yin *et al.* (2011) reported that the *csld3* mutant showed a 31% reduction in L7 root length, whereas the triple-mutant *csld2/csld3/csld5* had a 70% reduction in L7 root length, and they suggested that disruption of *CSLD3* may lead to a lower rate of cell division whereas both cell division and cell elongation may be disrupted in the triple-mutant. We only detected a 15% reduction in L6 root length, probably due to different plant culture conditions. In the present study, the *csld3* mutant showed reduced root-cell length by 5% (Fig. 2), but the transgenic line overexpressing *CSLD3* showed significantly increased cell number and length, confirming that CSLD3 could affect both cell elongation and cell division. In addition, numerous studies have demonstrated that cellulose affects cell elongation and that mutations in primary wall cellulose synthase (CesAs) typically lead to reduced cell elongation and loss of anisotropic growth (Fagard *et al.*, 2000; Cano-Delgado *et al.*, 2003; Chen *et al.*, 2010, 2016; Hu *et al.*, 2017; Li *et al.*, 2017). Here, both *AtCSLD3*- and *GhCSLD3*-overexpressing lines exhibited significantly enhanced cellulose production and the *csld3-1* mutant had decreased cellulose production in both seedlings and mature plants, which might explain the increase in cell elongation. Despite the low levels of *GhCSLD3* expression in etiolated hypocotyls of cotton (Fig. 1A), the increased length of hypocotyls, roots, and root hairs in Arabidopsis that overexpressed *GhCSLD3* suggests that this gene may also affect cell elongation in cotton.

The products of the CSLD enzyme remain largely controversial. Previous studies have shown that mutations of *CSLD* genes lead to reduced xylan, mannan, or homogalacturonan polysaccharides (Bernal *et al.*, 2007; Li *et al.*, 2009; Verhertbruggen *et al.*, 2011; Yin *et al.*, 2011). However, several studies support a role of CSLD in cellulose synthesis (Doblin *et al.*, 2010; Park *et al.*, 2011). Here, we analysed the cell wall composition of both *CSLD3*-overexpressing lines and the *csld3-1* mutant in primary (young root) and secondary (mature stem) cell walls. Notably, only the cellulose levels were significantly increased in the *CSLD3*-overexpressing lines and, conversely, levels were reduced in the the *csld3-1* mutant (Fig. 10). By comparison, other wall polysaccharides showed more complex changes in both the overexpressing transgenic lines and the mutant (Supplementary Fig. S7), suggesting that CSLD3 may be mainly involved in cellulose biosynthesis with indirect impacts on other wall polysaccharides. In addition, our findings indicate that CSLD3 might not only provide *β*-1,4-linked glucan synthase activity during root-hair development as previously reported (Park *et al.*, 2011), but it may also promote cellulose synthesis in other tissues.

In conclusion, we propose a hypothetical model to show that the function of CSLD3 is essential for root-hair and root growth even if ethylene and phosphate levels are altered. However, in contrast, CSLD3-mediated hypocotyl elongation occurs largely independently of ethylene signaling (Fig. 11). Our data also provide potential connections between *CSLD3* and the two root hair-specific *expansin* genes *AtEXPA7* and *AtEXPA18* in the control of root-hair elongation induced by environmental and developmental cues, as reported previously (Cho and Cosgrove, 2002; Guo and Ecker, 2004; Grierson *et al.*, 2014; Hwang *et al.*, 2016; Song *et al.*, 2016).

## Supplementary data

Supplementary data are available at *JXB* online.

Table S1. Primers used for *GhCSLD3* full-length cDNA cloning.

Table S2. Primers used for Q-PCR.

Table S3. Primers used for overexpression vector construction.

Fig. S1. Identification of the *GhCSLD3* gene.

Fig. S2. Phenotypes of the WT, *AtCSLD3*, and *GhCSLD3* complemented seedlings.

Fig. S3. Schematic diagram of RNAi screening (*i-ctr1* and *i-pdf1.2*) and the T-DNA insertion mutants (*t-ein2* and *t-pdf1.2*).

Fig. S4. Q-PCR analyses of gene expression levels using L6 seedlings grown on half-MS media.

Fig. S5. Comparison of ethylene responses among *pdf1.2* mutants and *CSLD3* complemented lines.

Fig. S6. Quantitative analyses of the *csld3-1* mutant treated with or without 5 μM ACC.

Fig. S7. Analysis of wall polysaccharides in 6-d-old light-grown roots and 7-week-old stems.

Supplemental Data

Supplemental Figures
