# Supplementary material for: AtCSLD3 and GhCSLD3 mediate root growth and cell elongation downstream of the ethylene response pathway in Arabidopsis
Source: J Exp Bot. 2017 Dec 14;69(5):1065–80. doi: 10.1093/jxb/erx470 (PMC6018909; doi:10.1093/jxb/erx470)

AtCSLD3 and GhCSLD3 mediate root growth and cell elongation downstream of the ethylene response pathway in *Arabidopsis*

Huizhen Hu, Ran Zhang, Shuchao Dong, Ying Li, Chunfen Fan, Yanting Wang, Tao Xia, Peng Chen, Lingqiang Wang, Shengqiu Feng, Staffan Persson, Liangcai Peng

Supplemental Table S1. Primers used for *GhCSLD3* full- length cDNA cloning.

Type	Sense/Antisense	Primer name	Primer sequence (5'-3')	Position (bp)
Middle fragment	sense	GhD33GSPL	CAGAGACAGAATAGGGAAGATGA	2051-2073
	antisense	GhD33GSPR	ACGGGGAATGGTGAGAGC	3121-3138
3' RACE	sense	HD33P1- out	GCCCTGAGGAAAATCGTGCTCTGAGAAT	2941-2968
	sense	HD33P2- in	TAAGACTGAGTGGGGACACCGTGTTG	3200-3225
	antisense	GhBC1- out	CAGTGAGCAGAGTGACGAGGA	
	antisense	GHBC1- in	CGAGGACTCGAGCTCAAGCT	
		QTAdaptor	CCAGTGAGCAGAGTGACGAGGACTCGAGCTC AAGCTTTTTTTTTTTTTTTTT	
5' RACE	sense	5'RACE -outer	CATGGCTACATGCTGACAGCCTA	
	sense	5'RACE- inner	CGCGGATCCACAGCCTACTGATGATCAGTCGATG	
	antisense	HD35P1- out	CAAGCAAGTTTTTCAACAGGGTAATCCG	1718-1745
	antisense	HD35P2- in	TTACCAGTAGGGTTGTTAAGGGTTGGTG	1593-1620
Full length	sense	D3-FW2	GAAAGAGTTTCGATTGTTCTG	369-389
	antisense	D3-RV	GAAGCATAAATATAGATGGCGATCC	4426-4450

Supplemental Table S2. Primers used for Q-PCR

Genes	Primer name	Primer sequence (5'-3')	TM (°C)	Length (bp)	
				cDNA	DNA
AtCSLD3	D3-RL	ATGGGTCCTGTTTATGTCGG	58	156	156
	D3-RR	AGATCGGTTTTCTTCTGGCA			
GhCSLD3	GhD3-1RL	GAGACAGAATAGGGAAGATGAACC	58	254	254
	GhD3-1RR	CACGAGAAACATAGACAAGCAAAG			
AtCTR1	57-RL	TTGTGGGAGCTTGCTACATTG	58	223	300
	57-RR	GGAGGAACCGCTGATTTGAT			
AtEIN2	EN-RL	TTTCATCGGTTCCTAACTGTGG	58	242	322
	EN-RR	TCTCTGGTGGCTCGCTGG			
AtPDF1.2	55-RL	TCACCCTTATCTTCGCTGCTC	58	220	300
	55-RR	TTAACATGGGACGTAACAGATACAC			
AtEXPA18	A18-RL	ACTATGGGTGGGGCTTGTGG	58	250	500
	A18-RR	AATGGACTCGTGGCGGATTA			
AtGAPDH	QGAPDH-F	GCAACATACGACGAAATCAAGAA	58	217	398
	QGAPDH-R	CGACACGAGAACTGTAACCCC			
GhUQB7	UBQ7QRT-F	GAAGGCATTCCACCTGACCAAC	58	250	250
	UBQ7QRT-R	CTTGACCTTCTTCTTCTTGTGCTTG			

Supplemental Table S3. Primers used for overexpression vector construction.

Type	Genes	Primer name	Primer sequence (5’-3’)	Restriction Enzyme cutting site	TM (°C)	Length of amplification (bp)
OE	*AtCSLD3*	OEAtD3-F	GGGGTACCATGGCGTCTAATAATCATTTCATG	kpnI	56	2628
		OEAtD3-R	GCTCTAGAATTTTGGCTCAACTATACTTGTGTGT	xbaI		
	GhCSLD3	OED3-F	CGGGGTACCGAAAGAGTTTCGATTGTTCTG	kpnI	57	3888
		OED3-R	GCTCTAGAGAAGCATAAATATAGATGGCGATCC	xbaI		
RNAi	*AtCTR1*	ct-L	GGATCCGAATTCATTTCTCCGATAGCCAGTC	EcoR I, Kpn I	52	626
		ct-R	AAGCTTGGTACCCTTAGCCACATCATAAGCC	BamHI, HindIII		
	AtPDF1.2	pdf-F	AAGCTTGAATTCGCTTCCATCATCACCCTTATCTTC	EcoR I, Kpn I	54	227
		pdf-R	ATCGATGGTACCCATGGGACGTAACAGATACACTTGT	HindIII, ClaI		
T-DNA	*AtPDF1.2*	pd1F	AGATAAGATGCACCGTCGATG		60	1000
		pd1R	ATTTGTTCGACGATGACGAAG			
	AtEIN2	f-LP	GTAACGCTGACGAAAAGCAAC		60	1000
		f-RP	CGCGTAAACTTTTCTCGAATG			
	AtCslD3	Csld3F	GAAGAAGAAAAGCCGTGTGC		60	1000
		Csld3R	AAGCCCTTTAGCGAATGGAT			
		LBb1.3	ATTTTGCCGATTTCGGAAC			

Supplemental Figure Legends

Supplemental Figure S1. Identification of the *GhCSLD3* gene. (A) Sequence alignment of *AtCSLD3* in cotton database. (B) Schematic diagram of *GhCSLD3* amplification using RACE-PCR. (C) Alignment of deduced amino acid sequences of GhCSLD3 and AtCSLD3. (D) Phylogenetic analyses of CSLD proteins in cotton, *Arabidopsis thaliana* and *Oryza sativa*.

Supplemental Figure S2. Phenotypes of the WT, *AtCSLD3* and *GhCSLD3* complemented seedlings. (A) L6 seedlings grown on 1/2 MS media. (B) Measurements of roots lengths of seedlings in (A). (C) and (D) Quantitative analyses of cell lengths in root EZ (C) and cell numbers in root MZ (D) of seedlings as shown in (A). Error bars represent mean ± SD (three biological replicates), $n \geq 50$ in (B), $n \geq 30$ in (C) and (D) seedlings were measured in each replicate. LSD test was used for multiple comparisons ($P < 0.01$). Scale bar indicates 5 mm in (A).

Supplemental Figure S3. Schematic diagram of RNAi screening (*i-ctr1* and *i-pdf1.2*) and the T-DNA insertion mutants (*t-ein2* and *t-pdf1.2*).

Supplemental Figure S4. Q-PCR analyses of gene expression levels using L6 seedlings grown on half-MS media. (A) and (B) *AtCSLD3* gene expression levels in WT, *AtCSLD3* overexpression lines and *csld3-1* mutant. (C) and (D) *GhCSLD3* gene expression levels in WT, *GhCSLD3* overexpression lines and *csld3-1* mutant. (E-G) *AtCTR1*, *AtEIN2* and *AtPDF1.2* genes expression levels in the corresponding mutants. Error bars represent mean ± SD (three biological replicates). LSD test was used for multiple comparisons. Different letters above bars indicate that the means differ according to analysis of variance and LSD test ($P < 0.01$). Percentage value (%) calculated by subtraction against WT divided by WT.

Supplemental Figure S5. Comparison of ethylene responses among *pdf1.2* mutants and *CSLD3* complemented lines. (A-F) Morphological phenotypes of L6 seedlings

under various treatment. Seedlings grown on 1/2 MS media (A), adding 1 μM ACC (B), adding 5 μM ACC (C), P- media (D) and (E), P- co-supplied with 1 μM ACC (F). (G-I) Quantitative analyses of root lengths of seedlings as shown in (A-F). Error bars represent mean ± SD (three biological replicates), n ≥ 30 seedlings were measured in each replicate. LSD test was used for multiple comparisons. Different letters above bars indicate that the means differ according to analysis of variance and LSD test ($P < 0.01$). Scale bar indicates 2 mm in (A, B, D, F); 500 μm in (C) and (E).

Supplemental Figure S6. Quantitative analyses of the *csld3-1* mutant treated with or without 5 μM ACC. Measurement of root length (A), root hair density (B) and root hair length (C). Error bars represent mean ± SD (three biological replicates). LSD test was used for multiple comparisons. Different letters above bars indicate that the means differ according to analysis of variance and LSD test ($P < 0.01$). Percentage value (%) calculated by subtraction against WT divided by WT.

Supplemental Figure S7. Analyses of wall polysaccharides in 6-day-old light- grown roots and 7-week-old stems. (A) and (C) Immunofluorescent labeling of roots (A) and stems (C) using plant cell wall glycan-directed monoclonal antibodies (mAbs). CCRC-M93 antibody against xyloglucans, CCRC-M38 for de-esterified homogalacturonan, CCRC-M35 for rhamnogalacturonan I. CCRC-M149 and CCRC-M38 antibodies are labeling for xylan and de-esterified homogalacturonan respectively. Antibody distribution is shown in green. (B) and (D) Monosaccharide composition analyses of L6 roots (B) and mature stems (D). Values represent means ± SD (three biological replicates). LSD test was used for multiple comparisons. Different letters above bars indicate that the means differ according to analysis of variance and LSD test ($P < 0.01$). Scale bar indicates 100 μm in (A) and (C).

Hu *et al.*, Supplemental Figure S1

A

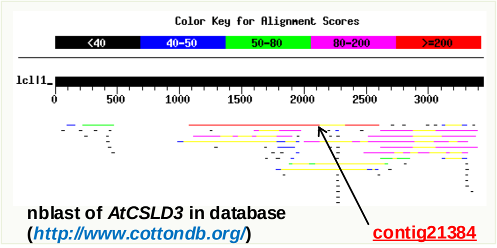

B

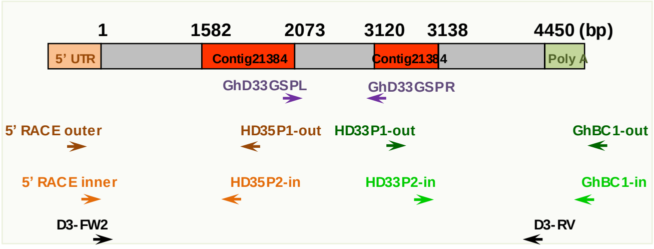

C

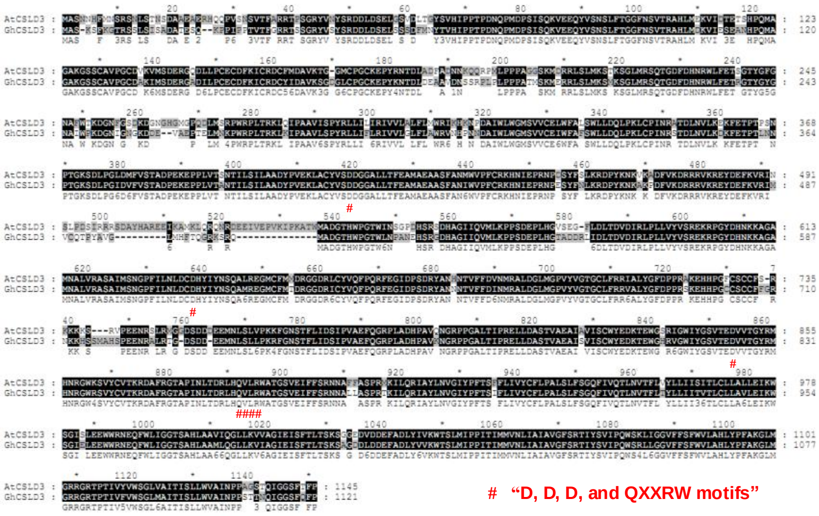

D

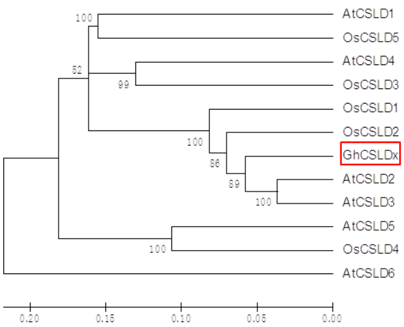

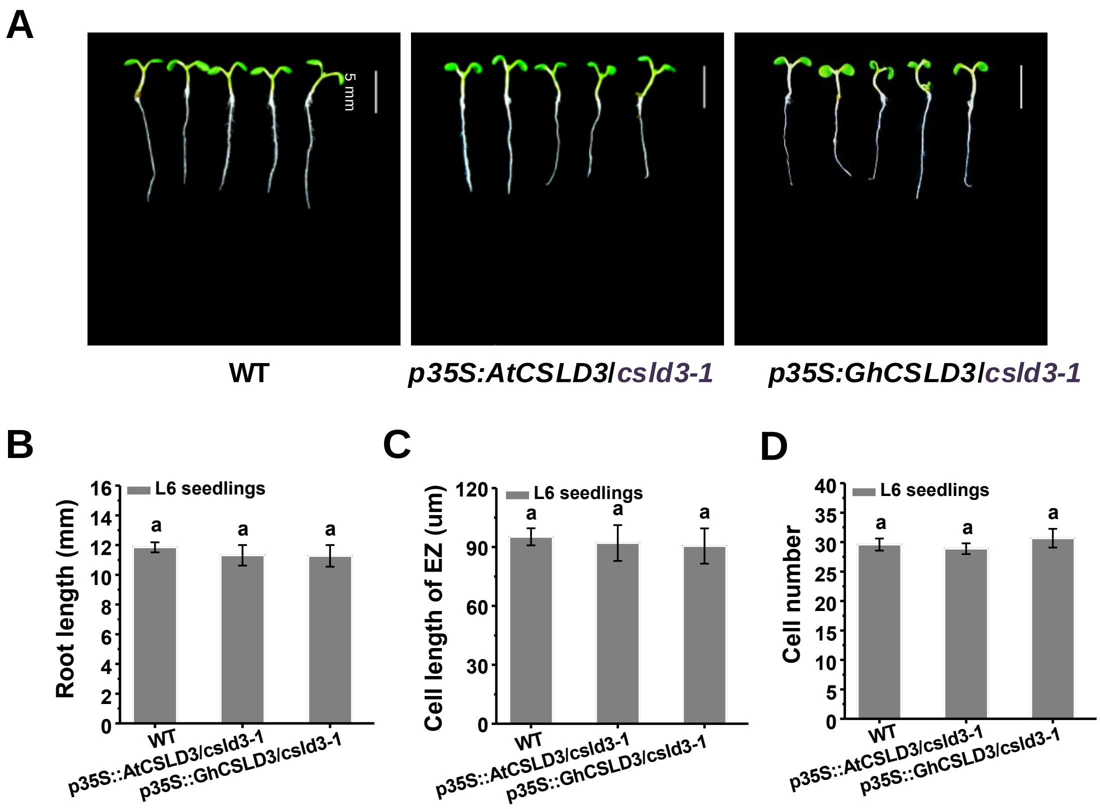
A
5 mm
WT
p35S:AtCSLD3/csld3-1
p35S:GhCSLD3/csld3-1
B
L6 seedlings
Root length (mm)
16
14
12
10
8
6
4
2
0
a
a
a
WT
p35S::AtCSLD3/csld3-1
p35S::GhCSLD3/csld3-1
C
L6 seedlings
Cell length of EZ (um)
120
90
60
30
0
a
a
a
WT
p35S::AtCSLD3/csld3-1
p35S::GhCSLD3/csld3-1
D
L6 seedlings
Cell number
40
35
30
25
20
15
10
5
0
a
a
a
WT
p35S::AtCSLD3/csld3-1
p35S::GhCSLD3/csld3-1

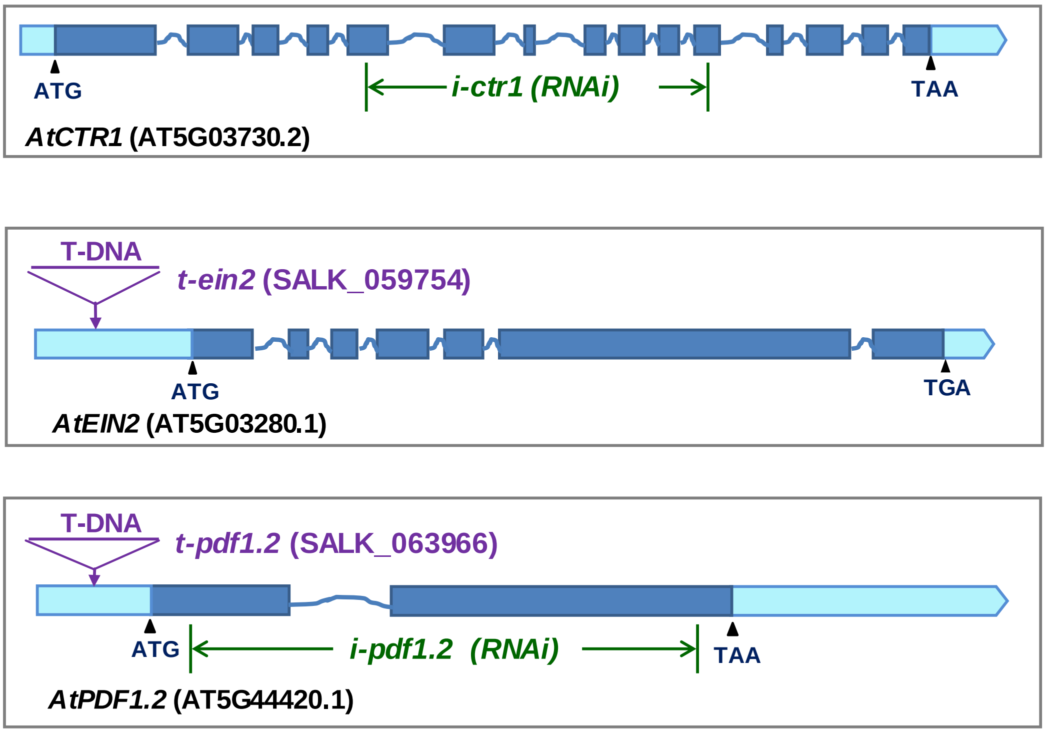

ATG
i-ctr1 (RNAi)
TAA
AtCTR1 (AT5G03730.2)
T-DNA
t-ein2 (SALK_059754)
ATG
TGA
AtEIN2 (AT5G03280.1)
T-DNA
t-pdf1.2 (SALK_063966)
ATG
i-pdf1.2 (RNAi)
TAA
AtPDF1.2 (AT5G44420.1)

Hu *et al.*, Supplemental Figure S4

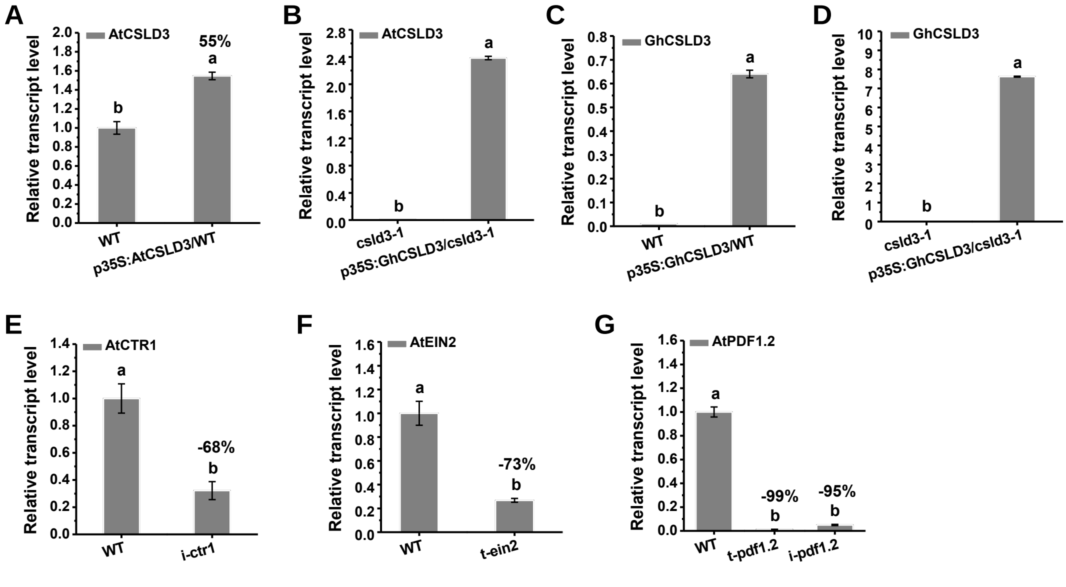

Hu *et al*., Supplemental Figure S5

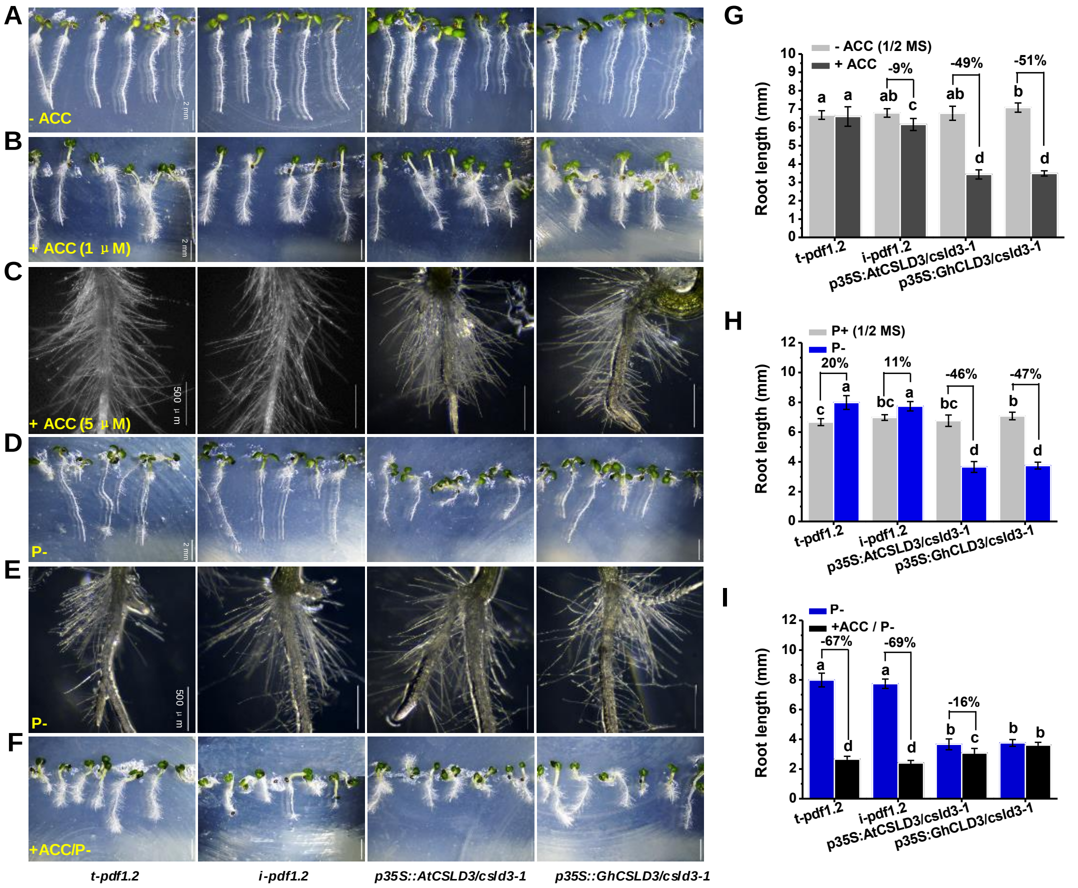

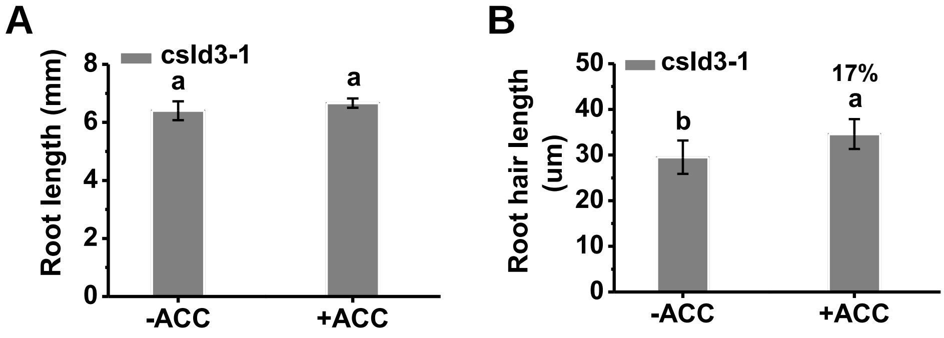
A
csld3-1
Root length (mm)
8
6
4
2
0
a
a
-ACC
+ACC
B
csld3-1
Root hair length (um)
50
40
30
20
10
0
b
17%
a
-ACC
+ACC

Hu *et al.*, Supplemental Figure S7

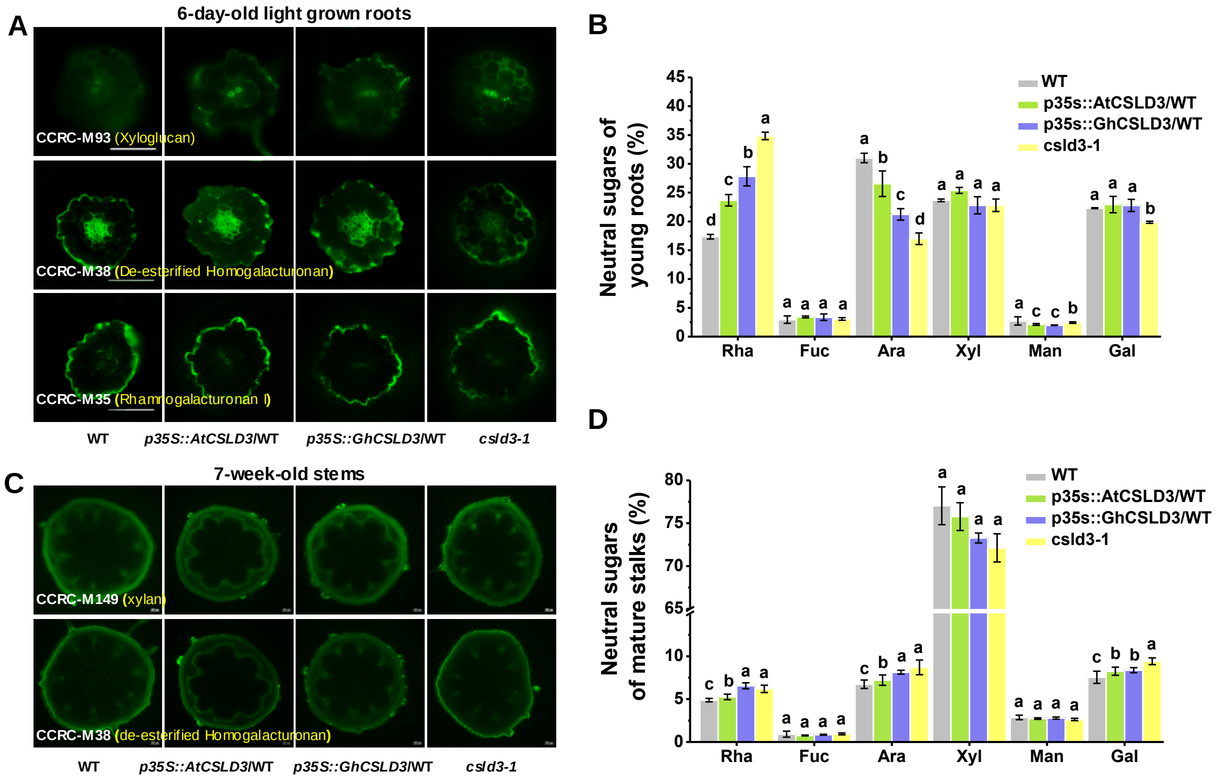

Supplement: Supplemental Data [file erx470_suppl_supplemental-data.pdf]
