# Supplementary material for: AtCSLD3 and GhCSLD3 mediate root growth and cell elongation downstream of the ethylene response pathway in Arabidopsis
Source: J Exp Bot. 2017 Dec 14;69(5):1065–80. doi: 10.1093/jxb/erx470 (PMC6018909; doi:10.1093/jxb/erx470)
Supplement: Supplemental Figures [file erx470_suppl_supplemental-figures.pptx]

## Slide 1
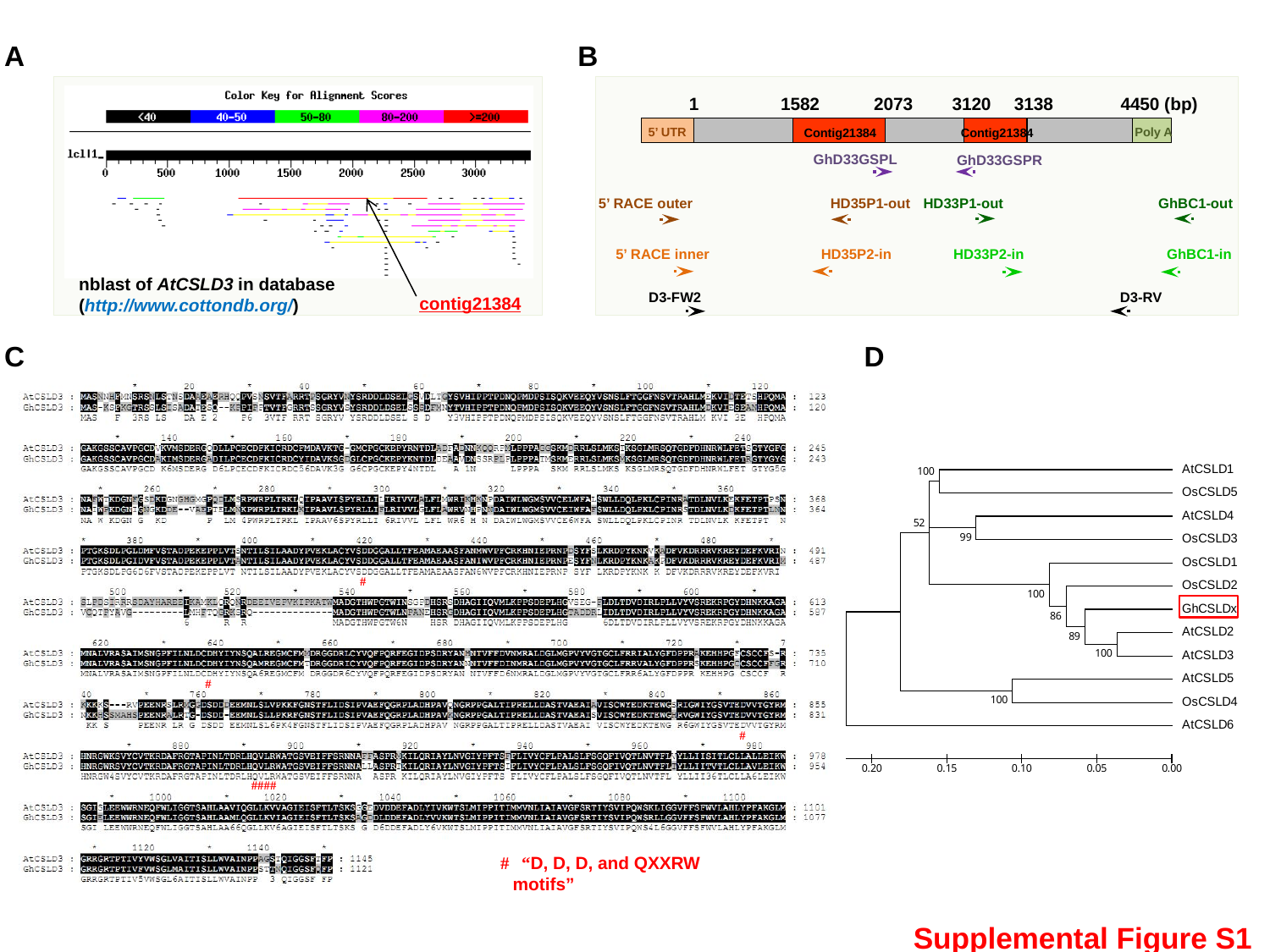

A
B
1
1582
2073
3120
3138
4450 (bp)
5’ UTR
Poly A
Contig21384
Contig21384
GhD33GSPL
GhD33GSPR
5’ RACE outer
HD35P1-out
HD33P1-out
GhBC1-out
5’ RACE inner
HD35P2-in
HD33P2-in
GhBC1-in
D3-FW2
D3-RV
nblast of AtCSLD3 in database (http://www.cottondb.org/)
contig21384
C
D
#
#
#
#
#
#
#
#
 “D, D, D, and QXXRW motifs”
# Supplemental Figure S1

## Slide 2
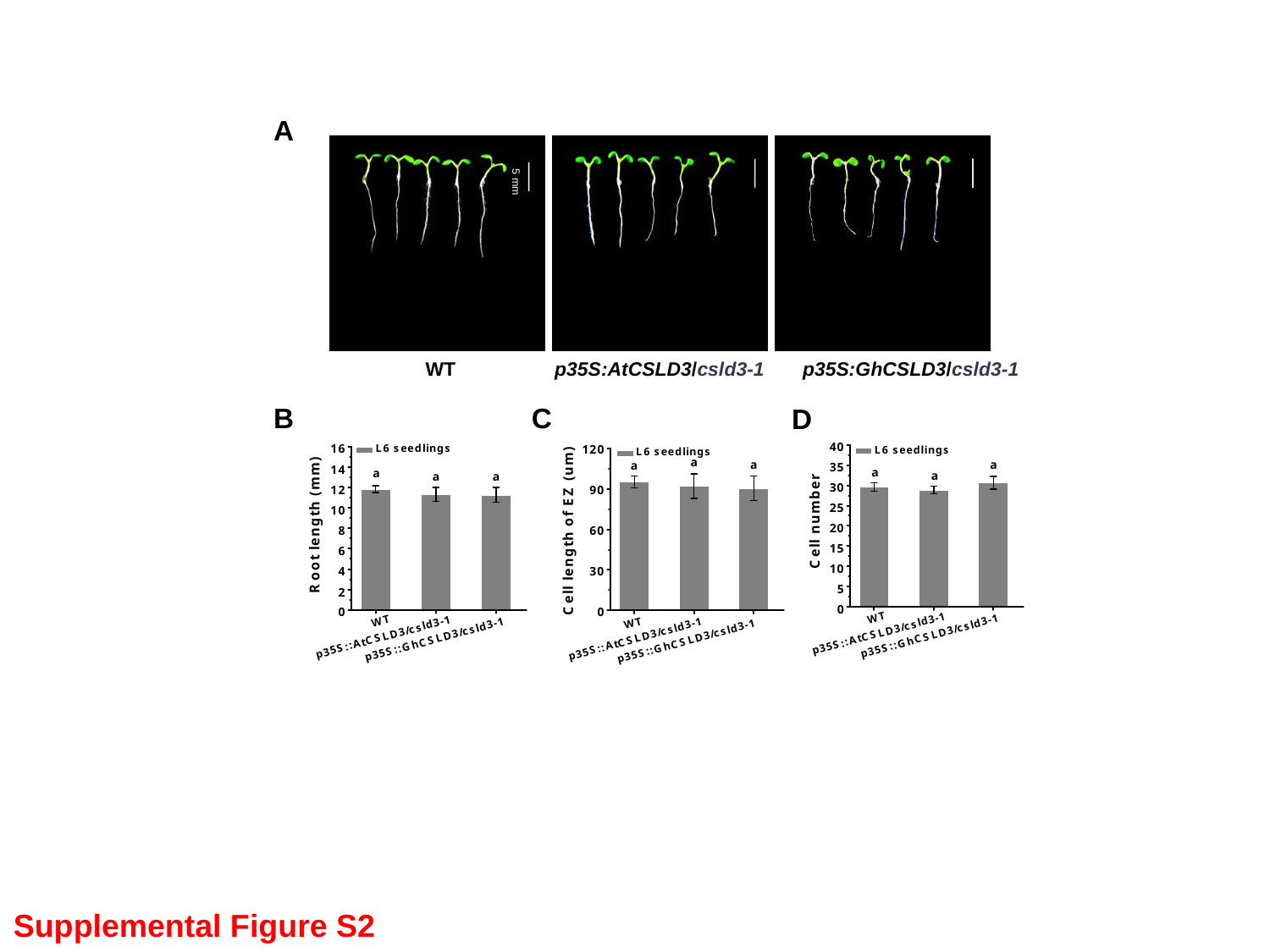

A
5 mm
 WT p35S:AtCSLD3/csld3-1 p35S:GhCSLD3/csld3-1
B
C
D
# Supplemental Figure S2

## Slide 3
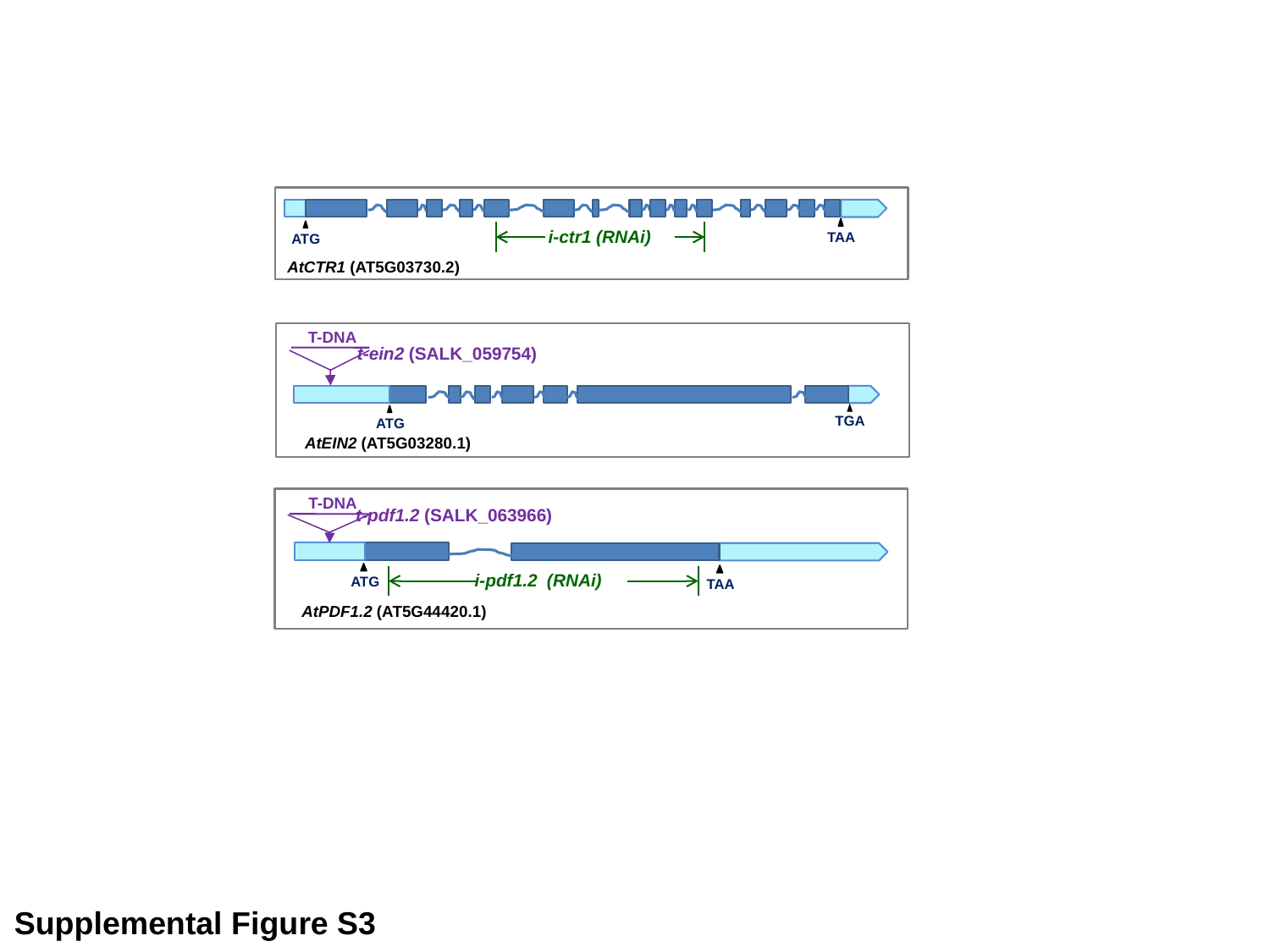

i-ctr1 (RNAi)
TAA
ATG
AtCTR1 (AT5G03730.2)
 T-DNA
t-ein2 (SALK_059754)
TGA
ATG
AtEIN2 (AT5G03280.1)
 T-DNA
t-pdf1.2 (SALK_063966)
i-pdf1.2 (RNAi)
ATG
TAA
AtPDF1.2 (AT5G44420.1)
# Supplemental Figure S3

## Slide 4
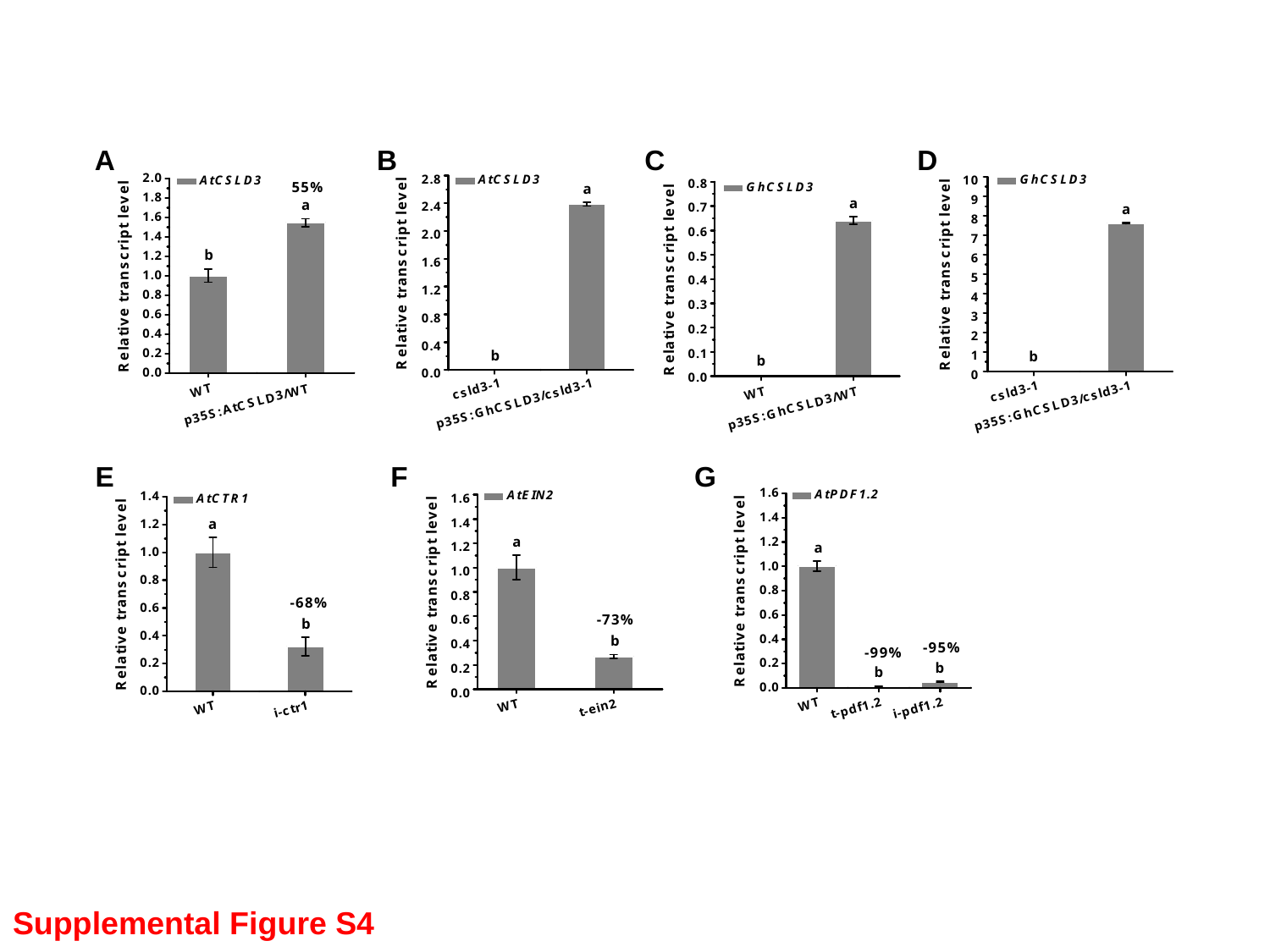

A
B
C
D
E
F
G
# Supplemental Figure S4

## Slide 5
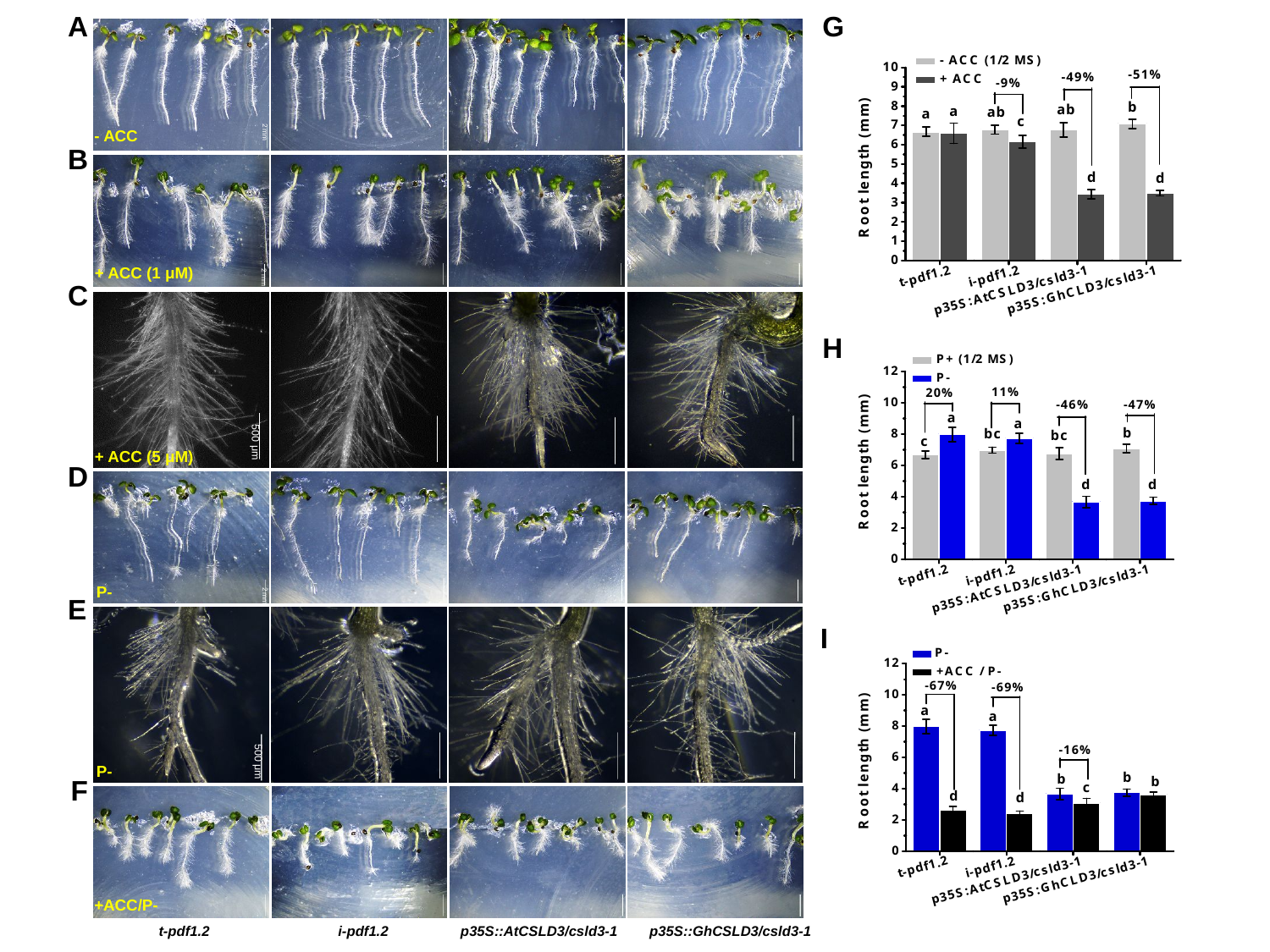

A
G
2 mm
- ACC
B
2 mm
+ ACC (1 μM)
C
H
500 μm
+ ACC (5 μM)
D
2 mm
P-
E
I
500 μm
P-
F
+ACC/P-
t-pdf1.2 i-pdf1.2 p35S::AtCSLD3/csld3-1 p35S::GhCSLD3/csld3-1

## Slide 6
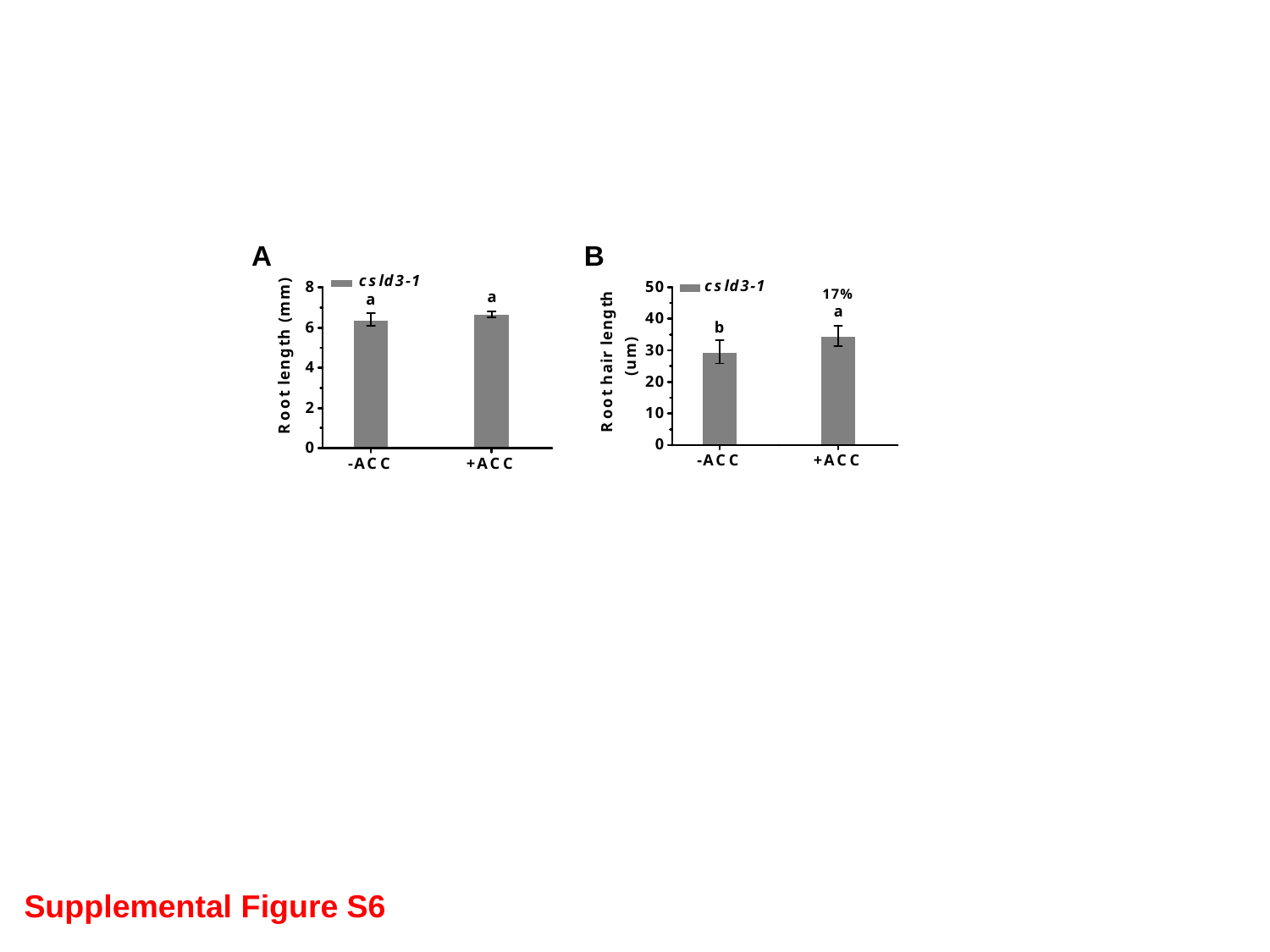

A
B
# Supplemental Figure S6

## Slide 7
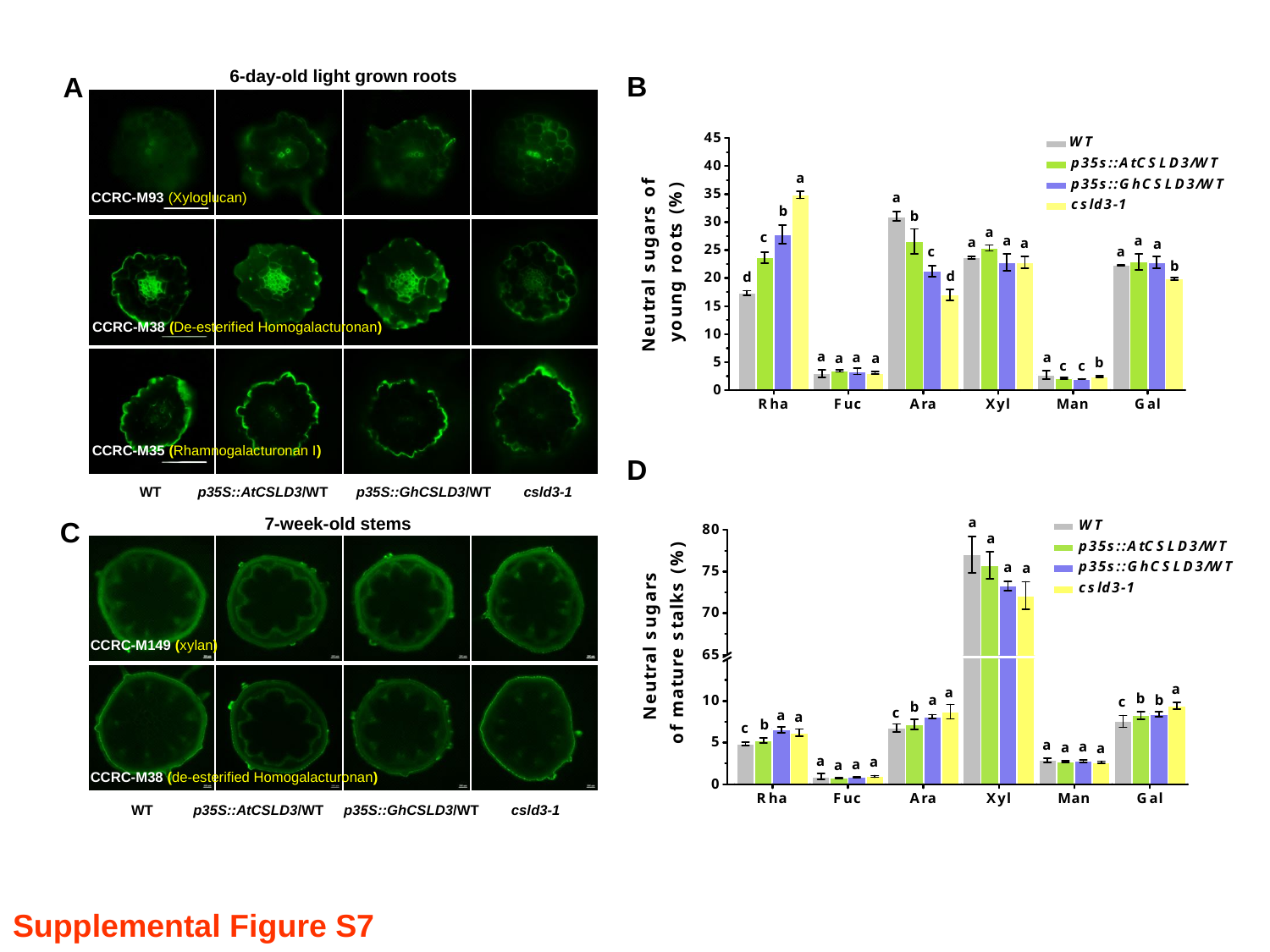

50um
6-day-old light grown roots
B
A
CCRC-M93 (Xyloglucan)
CCRC-M38 (De-esterified Homogalacturonan)
CCRC-M35 (Rhamnogalacturonan I)
D
 WT p35S::AtCSLD3/WT p35S::GhCSLD3/WT csld3-1
7-week-old stems
C
CCRC-M149 (xylan)
CCRC-M38 (de-esterified Homogalacturonan)
 WT p35S::AtCSLD3/WT p35S::GhCSLD3/WT csld3-1
# Supplemental Figure S7

## Slide 8
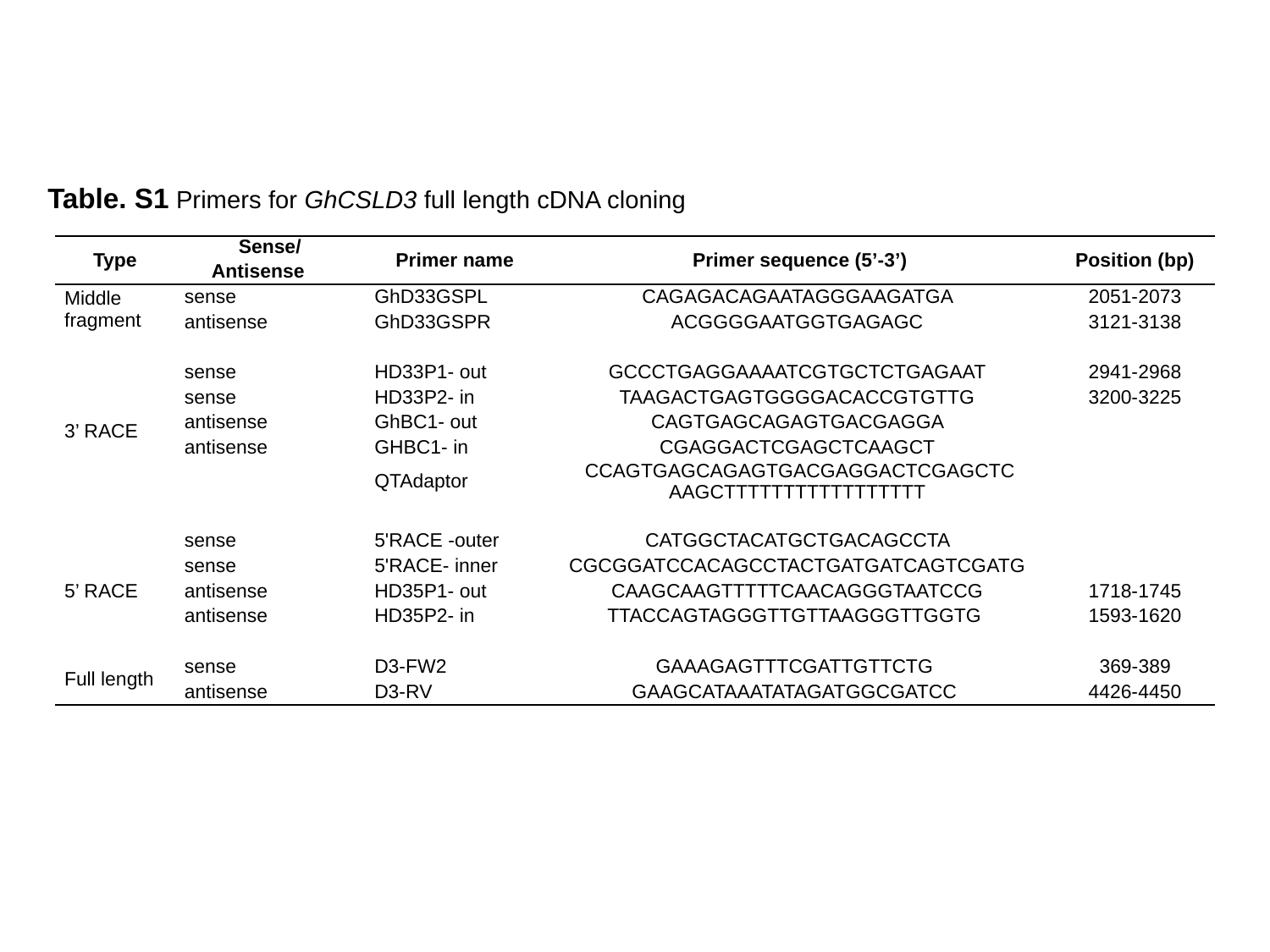

# Table. S1 Primers for GhCSLD3 full length cDNA cloning
| Type | Sense/Antisense | Primer name | Primer sequence (5’-3’) | Position (bp) |
| --- | --- | --- | --- | --- |
| Middle fragment | sense | GhD33GSPL | CAGAGACAGAATAGGGAAGATGA | 2051-2073 |
| | antisense | GhD33GSPR | ACGGGGAATGGTGAGAGC | 3121-3138 |
| | | | | |
| 3’ RACE | sense | HD33P1- out | GCCCTGAGGAAAATCGTGCTCTGAGAAT | 2941-2968 |
| | sense | HD33P2- in | TAAGACTGAGTGGGGACACCGTGTTG | 3200-3225 |
| | antisense | GhBC1- out | CAGTGAGCAGAGTGACGAGGA | |
| | antisense | GHBC1- in | CGAGGACTCGAGCTCAAGCT | |
| | | QTAdaptor | CCAGTGAGCAGAGTGACGAGGACTCGAGCTC AAGCTTTTTTTTTTTTTTTTT | |
| | | | | |
| 5’ RACE | sense | 5'RACE -outer | CATGGCTACATGCTGACAGCCTA | |
| | sense | 5'RACE- inner | CGCGGATCCACAGCCTACTGATGATCAGTCGATG | |
| | antisense | HD35P1- out | CAAGCAAGTTTTTCAACAGGGTAATCCG | 1718-1745 |
| | antisense | HD35P2- in | TTACCAGTAGGGTTGTTAAGGGTTGGTG | 1593-1620 |
| | | | | |
| Full length | sense | D3-FW2 | GAAAGAGTTTCGATTGTTCTG | 369-389 |
| | antisense | D3-RV | GAAGCATAAATATAGATGGCGATCC | 4426-4450 |

## Slide 9
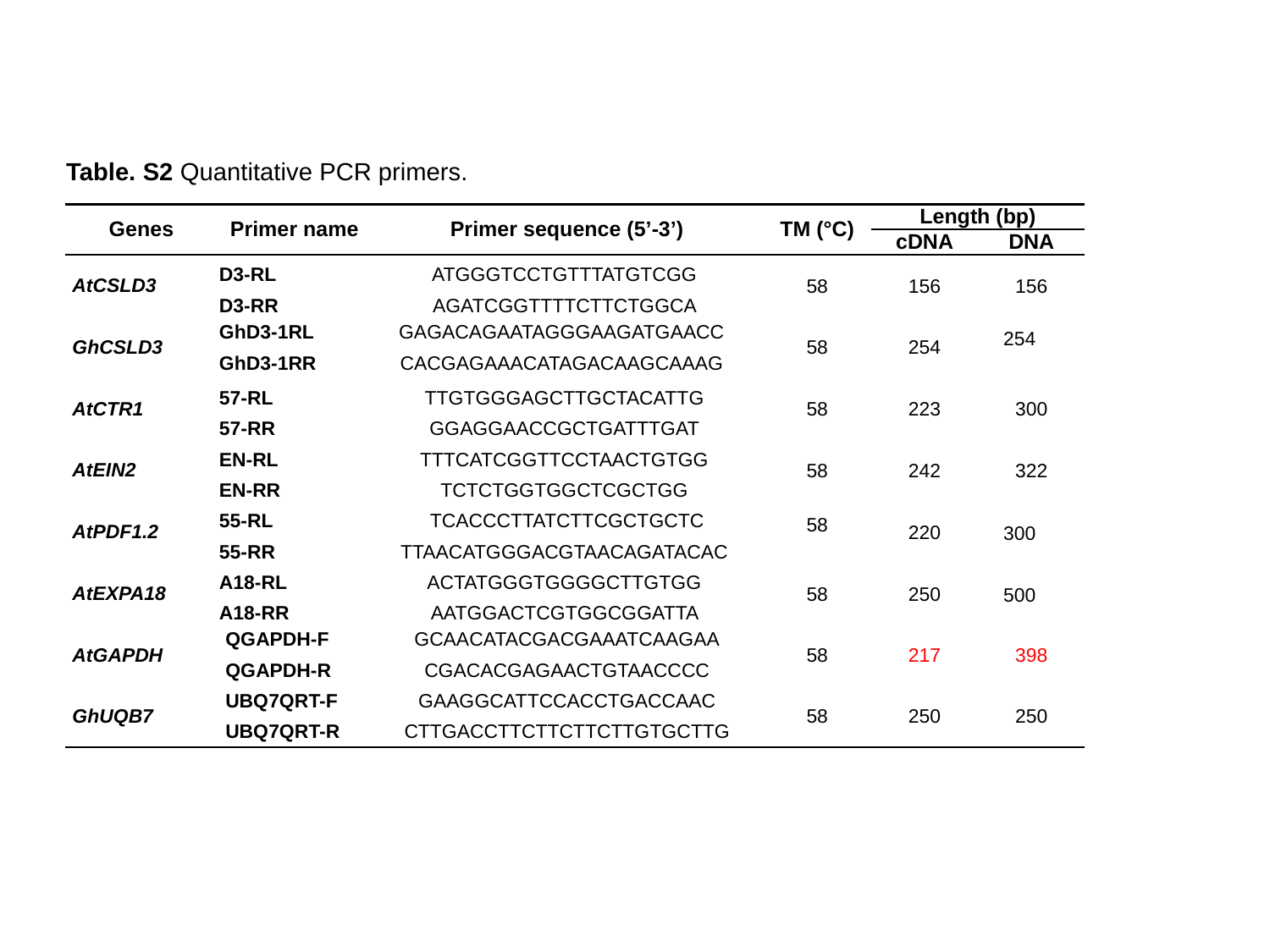

# Table. S2 Quantitative PCR primers.
| Genes | Primer name | Primer sequence (5’-3’) | TM (°C) | Length (bp) | |
| --- | --- | --- | --- | --- | --- |
| | | | | cDNA | DNA |
| AtCSLD3 | D3-RL | ATGGGTCCTGTTTATGTCGG | 58 | 156 | 156 |
| | D3-RR | AGATCGGTTTTCTTCTGGCA | | | |
| GhCSLD3 | GhD3-1RL | GAGACAGAATAGGGAAGATGAACC | 58 | 254 | 254 |
| | GhD3-1RR | CACGAGAAACATAGACAAGCAAAG | | | |
| AtCTR1 | 57-RL | TTGTGGGAGCTTGCTACATTG | 58 | 223 | 300 |
| | 57-RR | GGAGGAACCGCTGATTTGAT | | | |
| AtEIN2 | EN-RL | TTTCATCGGTTCCTAACTGTGG | 58 | 242 | 322 |
| | EN-RR | TCTCTGGTGGCTCGCTGG | | | |
| AtPDF1.2 | 55-RL | TCACCCTTATCTTCGCTGCTC | 58 | 220 | 300 |
| | 55-RR | TTAACATGGGACGTAACAGATACAC | | | |
| AtEXPA18 | A18-RL | ACTATGGGTGGGGCTTGTGG | 58 | 250 | 500 |
| | A18-RR | AATGGACTCGTGGCGGATTA | | | |
| AtGAPDH | QGAPDH-F | GCAACATACGACGAAATCAAGAA | 58 | 217 | 398 |
| | QGAPDH-R | CGACACGAGAACTGTAACCCC | | | |
| GhUQB7 | UBQ7QRT-F | GAAGGCATTCCACCTGACCAAC | 58 | 250 | 250 |
| | UBQ7QRT-R | CTTGACCTTCTTCTTCTTGTGCTTG | | | |

## Slide 10
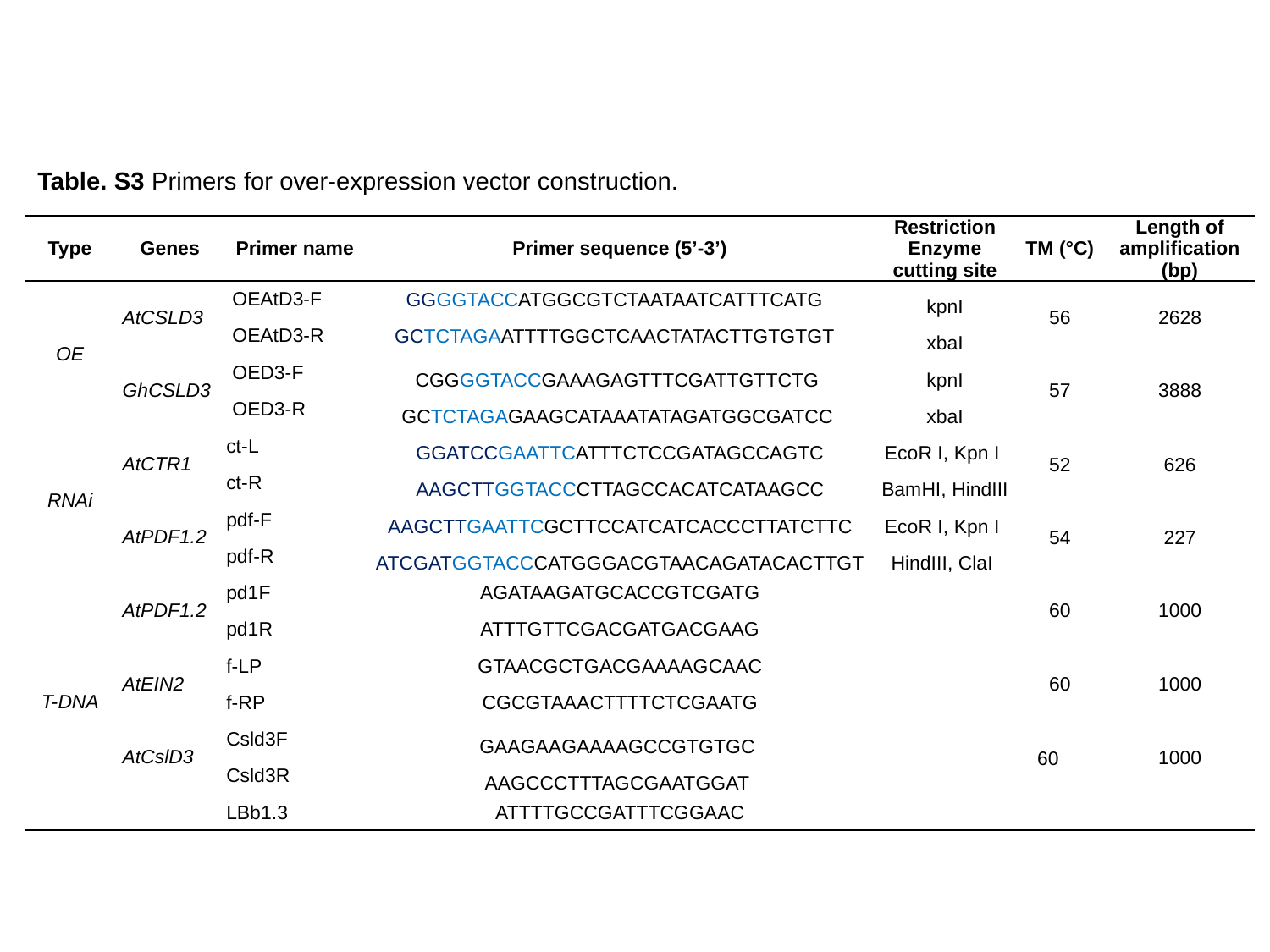

# Table. S3 Primers for over-expression vector construction.
| Type | Genes | Primer name | Primer sequence (5’-3’) | Restriction Enzyme cutting site | TM (°C) | Length of amplification (bp) |
| --- | --- | --- | --- | --- | --- | --- |
| OE | AtCSLD3 | OEAtD3-F | GGGGTACCATGGCGTCTAATAATCATTTCATG | kpnI | 56 | 2628 |
| | | OEAtD3-R | GCTCTAGAATTTTGGCTCAACTATACTTGTGTGT | xbaI | | |
| | GhCSLD3 | OED3-F | CGGGGTACCGAAAGAGTTTCGATTGTTCTG | kpnI | 57 | 3888 |
| | | OED3-R | GCTCTAGAGAAGCATAAATATAGATGGCGATCC | xbaI | | |
| RNAi | AtCTR1 | ct-L | GGATCCGAATTCATTTCTCCGATAGCCAGTC | EcoR I, Kpn I | 52 | 626 |
| | | ct-R | AAGCTTGGTACCCTTAGCCACATCATAAGCC | BamHI, HindIII | | |
| | AtPDF1.2 | pdf-F | AAGCTTGAATTCGCTTCCATCATCACCCTTATCTTC | EcoR I, Kpn I | 54 | 227 |
| | | pdf-R | ATCGATGGTACCCATGGGACGTAACAGATACACTTGT | HindIII, ClaI | | |
| T-DNA | AtPDF1.2 | pd1F | AGATAAGATGCACCGTCGATG | | 60 | 1000 |
| | | pd1R | ATTTGTTCGACGATGACGAAG | | | |
| | AtEIN2 | f-LP | GTAACGCTGACGAAAAGCAAC | | 60 | 1000 |
| | | f-RP | CGCGTAAACTTTTCTCGAATG | | | |
| | AtCslD3 | Csld3F | GAAGAAGAAAAGCCGTGTGC | | 60 | 1000 |
| | | Csld3R | AAGCCCTTTAGCGAATGGAT | | | |
| | | LBb1.3 | ATTTTGCCGATTTCGGAAC | | | |
